# Method feasibility for cross-species testing, qualification, and validation of the Filovirus Animal Nonclinical Group anti-Ebola virus glycoprotein immunoglobulin G enzyme-linked immunosorbent assay for non-human primate serum samples

**DOI:** 10.1371/journal.pone.0241016

**Published:** 2020-10-29

**Authors:** Nancy A. Niemuth, Thomas L. Rudge, Karen A. Sankovich, Michael S. Anderson, Nicholas D. Skomrock, Christopher S. Badorrek, Carol L. Sabourin

**Affiliations:** 1 Battelle Biomedical Research Center, West Jefferson, Ohio, United States of America; 2 Contract Support for the U.S. Department of Defense (DOD) Joint Program Executive Office for Chemical, Biological, Radiological, and Nuclear Defense (JPEO-CBRND) Joint Project Manager for Chemical, Biological, Radiological, and Nuclear Medical (JPM CBRN Medical), Fort Detrick, Maryland, United States of America; Tulane University, UNITED STATES

## Abstract

An anti-Zaire Ebola virus (EBOV) glycoprotein (GP) immunoglobulin G (IgG) enzyme linked immunosorbent assay (ELISA) was developed to quantify the serum levels of anti-EBOV IgG in human and non-human primate (NHP) serum following vaccination and/or exposure to EBOV. This method was validated for testing human serum samples as previously reported. However, for direct immunobridging comparability between humans and NHPs, additional testing was warranted. First, method feasibility experiments were performed to assess cross-species reactivity and parallelism between human and NHP serum samples. During these preliminary assessments, the goat anti-human IgG secondary antibody conjugate used in the previous human validation was found to be favorably cross-reactive with NHP samples when tested at the same concentrations previously used in the validated assay for human sample testing. Further, NHP serum samples diluted in parallel with human serum when tested side-by-side in the ELISA. A subsequent NHP matrix qualification and partial validation in the anti-GP IgG ELISA were performed based on ICH and FDA guidance, to characterize assay performance for NHP test samples and supplement the previous validation for human sample testing. Based on our assessments, the anti-EBOV GP IgG ELISA method is considered suitable for the intended use of testing with both human and NHP serum samples in the same assay for immunobridging purposes.

## Introduction

The filoviruses (family Filoviridae) from the genera Ebolavirus and Marburgvirus are etiologic agents of sporadic viral hemorrhagic fever outbreaks in humans with high mortality rates. An unprecedented outbreak of Ebola virus (EBOV; species Zaire ebolavirus) disease that began in Guinea during December 2013 [1] subsequently spread into neighboring West African countries of Sierra Leone and Liberia, prompting the World Health Organization (WHO) to declare the epidemic a public health emergency of international concern [2]. The outbreak ended in June 2016 with more than 28,600 cases and 11,325 deaths [3]. The recently ended outbreak in the Democratic Republic of Congo resulted in an overall case fatality ratio of 66% with 2287 fatal cases as of [4].

The EBOV glycoprotein (GP) is expressed on the exterior of the viral particle, is required for virus binding and entry into the cytoplasm of susceptible host cells and is a primary target for neutralizing and protective antibodies [5–11]. During the 2014–2016 EBOV disease outbreak in Sierra Leone, higher levels of EBOV anti-GP-specific IgG at one week after the onset of symptoms were correlated with survival in 65 confirmed cases [12]. Therefore, an efficacious vaccine candidate likely would require strong induction of GP-specific antibodies. The recently licensed vaccine [13] and other published vaccines present the EBOV GP as an antigen and induce anti-EBOV GP antibodies in non-human primates [14–19] and humans [20–36].

The approval of future vaccines against filoviruses will likely require extensive animal model testing and licensure under the FDA Animal Rule in the absence of a large outbreak. The model used to establish the efficacy and confirm the dose of ERVEBO^®^ was the cynomolgus macaque challenge model. The application of this model to the analysis of vaccines against other filoviruses will require correlates of immunity that are predictive of clinical benefit using biomarkers to bridge from the effective dose in the nonhuman primate model to the immune response elicited by the vaccine in placebo-controlled human trials.

Species-neutral immunological methods are ideal for bridging data between humans and animal models. The Filovirus Animal Nonclinical Group (FANG) [37] anti-EBOV GP IgG ELISA was developed and validated for testing human serum samples using a human reference standard (RS) and secondary antibody conjugate as previously reported [38] and shown to produce consistent results in multiple laboratories when testing human samples [39]. In this report we show that the anti-EBOV GP IgG ELISA using human RS and secondary antibody conjugate can be used to test NHP serum samples, which makes it useful for direct immunobridging applications. Method feasibility experiments assessed human and NHP RSs and secondary antibody conjugates in a head-to-head cross-species comparison of reactivity and parallelism using the same human and NHP test samples (TS). Feasibility results indicated that goat anti-human secondary antibody conjugate was cross reactive with NHP serum samples at similar concentrations used in the previous method validation for human serum sample testing. Additional side-by-side testing between human and NHP serum samples further supported that NHP serum samples appear to dilute in parallel to human serum. Once cross-species reactivity and parallelism were established, matrix qualification and partial validation were performed to characterize assay accuracy, precision, limits of quantification, and limit of detection for NHP samples when tested in the anti-EBOV GP IgG ELISA using a human RS and secondary antibody conjugate.

## Materials and methods

The validated human anti-EBOV GP IgG ELISA [38] was used for all testing, with exceptions described below for method feasibility experiments, using the critical reagents shown in Table 1.

**Table 1:** Critical reagents used in the anti-EBOV GP IgG ELISA.

**Reagent**	**Virus/Variant**	**Supplier**	**Lot Number**	**Coating Amount per Well**
rGP	EBOV Kikwit	Advanced Bioscience Laboratories; Rockville, MD	17OCT13	50 ng
**Reagent**	**Lot Number**	**Species**	**Concentration**^1^ **(ELISA Units/mL)**	**Starting Plate Dilution**
Reference Standard	BMIZAIRE102	Human	1009	1:100.9
	BMIZAIRE108^2^	Human	876	1:87.6
	BMIZAIRE007	NHP	1104	1:110.4
Quality Control High	BMIZAIRE103	Human	547.00	1:50
	BMIZAIRE110^2^	Human	490.37	1:50
	BMIZAIRE004	NHP	452.57	1:50
Quality Control Low	BMIZAIRE104	Human	148.40	1:50
	BMIZAIRE109^2^	Human	182.28	1:50
	BMIZAIRE005	NHP	36.20	1:50
Negative Control	BMI530	Human	0.00	1:50
	BMI298	NHP	0.00	1:50
	BMI300*	NHP	0.00	1:50
Conjugate	Jackson ImmunoResearch (Cat. No. 109-035-098)	Human	118460	1:4,000–1:16,000
	Fitzgerald (Cat. No. 43R-IG020HRP)	NHP	BMIC13011515	1:26,000

^1^Concentrations for human and NHP RS and QC lots were established via testing in species-specific assays and are comparable within species, but not directly comparable between species.

^2^Used in NHP Partial Validation.

### Method feasibility experiment

A preliminary experiment was performed to confirm that the human conjugate concentration used in the assay was acceptable for use with NHP samples. A total of four plates were prepared by two test operators (two plates per test operator, both on the same test day). The NHP RS BMIZAIRE007 was diluted in ELISA Diluent 1:2 across the plate from Column 1 through Column 11 in each row of the plates using the normal starting dilution of 1:110.4 for this lot in the qualified assay using NHP reagents. The negative control (NC; lot BMI298) was tested using a starting dilution of 1:50 in Column 12 of the plate (S1 Fig). Human conjugate (lot 118460, which is the current lot used in the anti-EBOV GP IgG ELISA) was used in each of seven rows, with concentrations ranging from 1:4000 to 1:16,000 and no conjugate (ELISA Diluent only) in row H (S2 Fig). Experimental data was visually evaluated to determine whether an NHP RS generated a response curve while background remained low to the human conjugate before proceeding with sample testing.

To evaluate the feasibility of using a human RS and a goat anti-human secondary antibody conjugate for testing NHP serum samples, a cross-reactive study was designed to look at both human and NHP RSs and secondary antibody conjugates in the presence of the same human and NHP TSs. A set of 12 human and 12 NHP TSs (S1 Table) were tested in the ELISA using (1) human RS and human conjugate and (2) NHP RS and NHP conjugate. The human samples were completely de-identified before researchers accessed the samples. The experiment used a modified plate layout similar to the standard layout [38]. The modified plate layout includes an RS, negative control (NC; species-matched to RS), four human TSs, four NHP TSs, and both the human and NHP quality control (QC) samples on each plate (S3 Fig and Table 1). Species-specific system suitability criteria were applied to the RS, NC, and same-species QC samples. TS acceptance criteria were applied to dilutions within the RS range. Over two months, seven test operators analyzed the 12 human and 12 NHP TSs, along with human and NHP QC, on a total of 22 passing plates. TSs were selected from ongoing clinical [38] and preclinical studies, to include multiple vaccine products. High concentration samples were required for the parallelism evaluation.

Parallelism between TSs and the RS on the same plate was initially evaluated using the assay TS acceptance criteria based on the Plikaytis method [40]. Test samples were considered to be parallel to the RS on the same plate if the %CV of the anti GP IgG concentration (ELISA units/mL), using at least three dilutions, was less than 20%. The proportion of the test samples that met the %CV criterion and 90% confidence intervals were calculated. A high proportion of TSs meeting this criterion is indicative that the TS and RS are parallel. In this assay, QC samples are diluted similarly to TSs; therefore, for the purposes of this evaluation, QCs were treated as TSs and included in the proportion of samples that met the %CV criterion. TSs that failed for reasons other than %CV were excluded from the evaluation. For example, samples that needed to be retested at a different starting dilution were excluded because multiple dilutions were masked. All plates were included, provided that the RS and NC criteria were met.

Additionally, parallelism was evaluated by fitting a four-parameter logistic (4PL) regression model with sample-specific EC_50_ to the mean OD values for RS, TS, and QC samples on each plate against the base-10 logarithm of the relative concentration. The model assumed that the RS, TS, and QC samples had the same upper and lower asymptotes and slope parameters but allowed the EC_50_ concentration to vary depending on sample. As only six concentrations were tested for the TS and QC samples, separate 4PL models could not be fit to individual samples for direct comparisons of the steepness parameter. Therefore, to determine parallelism, the overall fit of the model was evaluated by calculating standardized residuals for the model on each plate and visually assessing the standardized residuals across all plates.

### NHP matrix qualification procedure

Background levels of naïve NHP sera in the human anti-GP IgG ELISA were used to determine the minimum starting dilution for test samples and to calculate a prediction interval for naïve samples, which was used to establish acceptance criteria for the NHP NC. One hundred fifty (150) naïve NHP serum samples were tested in the ELISA at starting dilutions of 1:25, 1:50, and 1:100; each sample was analyzed twice in duplicate (four OD values) at each dilution and the mean of the duplicate OD values was used as the endpoint in the statistical analysis. An analysis of variance (ANOVA) was fitted to the mean OD values with a single fixed effect for starting dilution (1:25, 1:50, and 1:100). Studentized residuals in excess of three in absolute value were considered potential outliers. Fourteen potential outliers were identified from 4 test samples. Investigation showed that the outlier values comprised more than half the data for these test samples and were spread across all three starting dilutions. All results for these four test samples were removed so that these test samples would not influence the limit of background cutoff value.

Five positive NHP immune serum samples (S2 Table) tested against the human RS in preliminary screening (3,600 to 36,000 ELISA units/mL) were used to prepare five Dilutional Linearity dilution sets for qualification testing. Each parent test sample was diluted into naïve NHP serum (Lot Number BMI300) to create the ten unique dilution samples for each dilution set. Some samples were tested at multiple starting dilutions; therefore 60 Qualification Test Samples (QTSs, QTS#1—#60) were prepared (S3 Table) and shared among the test operators.

Testing of the Dilutional Linearity QTSs (QTS#1—#60) occurred using specified layouts by three test operators over three days with each operator running up to a maximum of five plates per day. Each QTS was tested at least two times by each test operator. However, some QTSs were tested multiple times on the same plate (repeatability) and some were tested multiple times by the same test operator on the same day but on different plates (intermediate precision). Additionally, for each of the five dilution sets, one sample was tested at multiple starting dilutions to evaluate starting dilution effect. Each operator prepared their own set of RS, QCs, NC, and QTSs on each testing day.

Any plates or samples that failed plate or test sample acceptance criteria [38] were repeated upon the completion of the first round of testing (three days of testing by all three test operators) preserving the intended levels of replication as much as possible. For each QTS, both concentration (ELISA units/mL) and endpoint titer were reported; only the concentration results are reported here.

A preliminary ANOVA model was fitted to the log_10_(concentration) values for purposes of identifying potential outliers. The model contained a fixed effect for QTS and specified a separate logarithmic mean and logarithmic standard deviation within each QTS. Studentized residuals about the mean concentrations were determined from the model. Studentized residuals in excess of three in absolute value were considered potential outliers. Three potential outliers were identified and investigated. No anomalies in the testing process or processing paperwork were found; therefore, all observations were included in the statistical analysis.

A mixed effects ANOVA model was fitted to all the passing concentration data. Fixed effects for log_10_(spike level), log_10_(starting dilution), test specimen, and two-way interactions among the fixed effects, and random effects for operator, day, plate nested within day and operator, and replicate (residual error) were included in the model; the response variable was log_10_(concentration). Predicted values of log_10_(concentration x spike) were determined for each QTS.

The LOD was estimated using logistic regression analysis as the lowest predicted concentration (from the ANOVA model) for which the probability of determining an estimate experimentally is at least 95%.

The percent total variability of the log-transformed values for each parent test sample and spike level was calculated and back-transformed to the observational scale. An arbitrary cutoff of 50% was used as the desired maximum percent total variability. A preliminary upper LOQ (ULOQ) was set as the maximum concentration measured with acceptable variability. The preliminary lower LOQ (LLOQ) was calculated as the median of the concentrations from the last dilution level with acceptable variability. Final LOQs were defined based on results from the dilutional linearity and precision assessments.

The accuracy of an analytical method describes the closeness of mean test results obtained by the method to the true value of the analyte. For this assay, accuracy was assessed via dilutional linearity, i.e., whether the assay can obtain results that are proportional to the concentration of antibody in the sample. Dilutional linearity was determined through comparison of the resulting value for a QTS to the spike level (1/dilution) for that sample when diluted in naïve sera. Under perfect dilutional linearity, the slope of the regression of log-transformed concentration to log-transformed spike level should be -1. A random coefficients regression model was fit to data for each parent test sample between the preliminary LOQs to relate the log-transformed ELISA concentration to the log-transformed final dilution of each test sample. The slope and corresponding 90% confidence interval for each QTS and the overall regression line across all ten parent test samples were calculated.

The precision of an analytical method describes the closeness of agreement of individual measures of an analyte when the procedure is applied repeatedly to multiple aliquots of a single homogeneous volume of biological matrix. The two types of precision are: (1) intra-run precision (Repeatability), which assesses precision during a single analytical run (a group of assays performed by one person using one set of equipment, close together in time); and (2) inter-run precision (Intermediate Precision), which measures the precision between different runs and incorporates operator-to-operator, day-to-day, and plate-to-plate variability.

The previously described mixed effects ANOVA model was refitted to QTSs with concentrations within the LLOQ and ULOQ. Variability associated with each of the random effects as well as repeatability, intermediate precision, and total assay variability were estimated using model-based percent CV. The percent CV for each source of variance was calculated as $100\times \sqrt{e^{\text{ln}{\left(10\right)}^{2}\times \sigma^{2}}-1}$ where *σ*^2^ is the model-estimated variance for the specific variance source. The percent CV associated with the model residual is an estimate of replicate observations of the same sample on the same plate evaluated by the same operator on a given day, and hence served to estimate the assay repeatability. The percent CV associated with the test day, operator, and plate effects served as an estimate for the intermediate precision of the assay. Total assay variability was estimated using all variance components from the model (both inter- and intra-run variability).

The final assay LOQs were defined as the preliminary LOQs so long as the assay appeared to be dilutionally linear and precise.

Matrix effects were evaluated by spiking one positive NHP serum sample (NHP RS, BMIZAIRE007) and one positive human serum sample (Human RS, BMIZAIRE102) into six naïve samples (NHP Negative Control BMI300, four individual naïve NHP serum samples, and ELISA Diluent) at six spike levels (S4 Table). The 72 samples (QTS#61- #132) were created once and shared among the test operators. Each QTS was tested two independent times (one operator over two days or two operators over one day or equivalent combination). Using BMI530 as the comparator, the % difference between the observed concentration and expected concentration was calculated for each of the 12 QTSs (2 samples—human and NHP x 6 spike levels) for each naïve sample matrix. The mean % difference across the five naive test samples and a corresponding 90% confidence interval on this mean were calculated for: (1) each test sample/spike level combination; and (2) overall across all test samples and spike levels.

Specificity was evaluated using five positive NHP serum samples and one naïve NHP serum sample that were pre-adsorbed with EBOV Kikwit rGP antigen (at 25 μg/mL and 10 μg/mL), CMV antigen (at 25 μg/mL), or ELISA Diluent (mock). Each test sample was treated for each of the four conditions—pre-adsorbed with EBOV Kikwit rGP antigen (at 25 μg/mL and 10 μg/mL), CMV (Cytomegalovirus) antigen (at 25 μg/mL), or ELISA Diluent (mock) (S5 Table). The 24 QTSs (QTS#133-#156) were tested two independent times (one operator over two days or two operators over one day or equivalent combination). For each test condition, the difference of the log-transformed mock concentration and log-transformed test concentration was calculated for each of the five test samples. The mean difference across the five test samples and lower 90% confidence bound on this mean were calculated and back-transformed to the observational scale to obtain a ratio of geometric means and 90% lower confidence bound. All concentrations for the naïve serum sample were reported as zero and thus not included in this analysis.

### NHP partial validation procedure

There were three categories of validation test samples (VTSs) comprising a total of 161 VTSs: Dilutional Linearity Samples, Matrix Effects/Selectivity Samples, and Real World (Incurred) Samples. These VTSs were made from a combination of 38 serum samples positive for anti-GP IgG and six that were naïve (S6 Table). The 161 VTSs were prepared once for each VTS and shared among the test operators.

Validation assays were conducted using explicit plate layouts that were tested by specific test operators on specific days over a two-month period. Each VTS was intended to be tested eight times (twice by each of the four operators), with some exceptions. For example, VTSs (37 total) that were part of repeatability (within plate) and intermediate precision (between-operator, between-day, between-plate) evaluation were tested 16 times. Any plates or samples that failed plate or test sample acceptance criteria were repeated upon the completion of the first round of testing, preserving the intended levels of replication as much as possible. Each VTS was tested the intended number of times except for 28 repeatability VTSs that were tested more than the designated 16 times due to repeat testing. For those samples, passing result(s) from both the original plate and repeat plate were included as part of the statistical analysis. For each VTS, there were two reported assay endpoints: concentration (ELISA units/mL) and endpoint titer. The analysis of both endpoints was carried out in a manner directly analogous to each other and the formal validation acceptance criteria were based on both endpoints. For concentration, analysis was carried out using log_10_(concentration) except where noted. For endpoint titers, analysis was carried out using log_10_(endpoint titer) except where noted. Throughout the text, the notation “concentration (endpoint titer)” or “log_10_(concentration) [log_10_(endpoint titer)]” is used to indicate that the same analysis was performed separately for both endpoints.

A preliminary ANOVA model was fitted to the log_10_(concentration) [log_10_(endpoint titer)] values for purposes of identifying potential outliers. The model contained a fixed effect for VTS. Studentized residuals about the mean concentrations (endpoint titers) were determined from the model. Studentized residuals in excess of three in absolute value were considered potential outliers. Several potential outliers were identified and investigated: two were marked as failing and excluded from the concentration analysis and one from the endpoint titer analysis.

A mixed effects ANOVA model was fitted to all the passing data from dilutional linearity samples separately by endpoint. Fixed effects for log_10_(spike level), log_10_(starting dilution), test specimen and two-way interactions among the fixed effects, and random effects for operator, day, plate tested within day and operator, and replicate (residual error) were included in the model; the response variable was log_10_(concentration). Predicted values of log_10_(concentration x spike) were determined for each VTS. This model was also used to assess precision (described in precision section below).

The LOD is the lowest predicted concentration (endpoint titer) for which the probability of determining an estimate of anti-GP IgG antibody activity is at least 95%. The limit of detection analysis was based on data from dilutional linearity samples with starting dilutions of 1:50 for which a concentration (endpoint titer) could be predicted in the mixed model analysis of variance. The LOD for each endpoint was estimated using logistic regression analysis as the lowest predicted concentration (endpoint titer) from the ANOVA model for which the probability of determining an estimate experimentally is at least 95%.

The percent total variability of the log-transformed values for each parent test sample and spike level was calculated and back-transformed to the observational scale. An arbitrary cutoff of 50% was used as the desired maximum percent total variability. A preliminary ULOQ was set as the maximum concentration measured with acceptable variability. The preliminary LLOQ was calculated as the median of the concentrations from the last dilution level with acceptable variability. Final LOQs were defined based on results from the dilutional linearity and precision assessments.

Dilutional linearity was assessed relative to spike level (1/dilution) and starting dilution. For spike level, a random coefficients regression model was fit to results between the preliminary LOQs to relate the log-transformed ELISA concentration (endpoint titer) to the log-transformed spike level of each VTS. The slope and corresponding 90% confidence interval for each VTS and the overall regression line across all parent test samples were calculated. An equivalence analysis was conducted (e.g., two one-sided test) to determine if the confidence interval was completely contained within the validation acceptance interval of (-1.20, -0.80).

For starting dilution, a random coefficients regression model was fit to data within the preliminary LOQs to relate the log-transformed ELISA concentration (endpoint titer) to the log-transformed starting dilution of each VTS. The slope and corresponding 90% confidence interval for each VTS and the overall regression line across all parent test samples were calculated. An equivalence analysis was conducted (e.g., two one-sided test) to determine if the confidence interval was completely contained within the validation acceptance interval of (-0.20, 0.20).

The dilution and real-world samples were used in the assessment of assay precision. Assessment of intermediate precision, repeatability, and total assay variability was done differently for the two endpoints due to the discrete and discontinuous nature of the endpoint titer.

For concentration, the previously described mixed effects ANOVA model was refitted to VTSs with concentrations within the LLOQ and ULOQ. Variability associated with each of the random effects as well as repeatability, intermediate precision, and total assay variability were estimated using model-based percent CV. The percent CV for each source of variance was calculated as $100\times \sqrt{e^{\text{ln}{\left(10\right)}^{2}\times \sigma^{2}}-1}$ where *σ*^2^ is the model-estimated variance for the specific variance source. The percent CV associated with the model residual is an estimate of replicate observations of the same sample on the same plate evaluated by the same operator on a given day, and hence served to estimate the assay repeatability (intra-run variability). The percent CV associated with the test day, operator, and plate effects served as an estimate for the intermediate precision of the assay (inter-run variability). Total assay variability was estimated using all variance components from the model (both inter- and intra-run variability).

Due to the discrete nature of the endpoint titer, the assay is considered precise and repeatable when a large proportion of results are within one dilution (e.g., within two-fold) of the median titer of the replicates across all plates. For intermediate precision of the endpoint titer, no more than 10% of the endpoint titers could be more than one dilution different from the median titer calculated from all plates over all test operators and days, for at least 80% of samples. For repeatability of the endpoint titer, no more than 10% of the endpoint titers could be more than one dilution different from the median titer on the sample plate, for at least 80% of repeatability samples.

The final assay LOQs were defined as the preliminary LOQs so long as the precision criteria were met and the dilutional linearity criteria were met for both spike level and starting dilution.

The selectivity analysis was restricted to VTS#101-136, which were based on three positive NHP serum samples spiked at two concentrations (1:5 and 1:50) into five individual naïve NHP serum samples and into one pooled naïve serum sample (BMI300). Using BMI300 as the comparator, the percent (fold) difference between the observed concentration (endpoint titer) and expected concentration (endpoint titer) were calculated for each of the 6 positive test samples (3 samples x 2 spike levels) for each naïve sample matrix. The mean percent (fold) difference across the five naive test samples and a corresponding 90% confidence interval on this mean were calculated. An equivalence analysis was conducted (e.g., two one-sided tests) to determine if the confidence interval was completely contained within the validation acceptance interval of (-35%, 35%) for concentrations and (0.34, 3.00) for endpoint titer.

## Results

### Method feasibility experiments

A dilution of 1:10,000 for human conjugate 118460 was selected as the conjugate dilution for testing NHP samples. OD values with this conjugate dilution had sufficient maximum OD values and sufficient depth of curve as BMIZAIRE007 (NHP RS) was diluted across the plate (S7 Table). Other dilutions tested in the 1:4,000 to 1:16,000 range also appeared suitable. The 1:10,000 dilution is used for testing human samples and is in the middle of the range of suitable dilutions for testing NHP samples.

Test samples were considered to be parallel to the RS on the same plate if the %CV of the anti-GP IgG concentration (ELISA units/mL), using at least three dilutions, was less than 20% (Plikaytis, *et al*., 1994). NHP samples tested on plates using human RS/conjugate met this %CV criterion 85.9% of the time, compared to 95.1% of the time for human TS on those plates (Table 2). NHP and human TS met the %CV criterion with similar rates (88.4 and 87.8%, respectively) when tested against NHP RS/conjugate (Table 2). Both human and NHP QC samples were included on each plate and were included in the parallelism assessment. The number of dilutions meeting the %CV criterion corresponds to the range over which parallelism is established; a 2-fold dilution series is used in the assay. A strong majority (74.3 to 98.7%) of TS met the %CV criterion over four to six dilutions, which indicates parallelism over an 8-fold to 32-fold range for both human and NHP TS for both the human and NHP RS/conjugate test conditions.

**Table 2:** Method feasibility study: Summary of success rates based upon %CV criteria and number of dilutions that met the %CV criteria.

RS/Conjugate used on Plate	TS	N Pass/N Tested	Pass (%)	Number of Dilutions that Met %CV Criteria			
				6 (32-fold)	5 (16-fold)	4 (8-fold)	3 (4-fold)
Human	Human	77/81	95.1%	6 (7.8)	26 (33.8)	26 (33.8)	19 (25.7)
	**NHP**^1^	**61/71**	**85.9%**	**1 (1.6)**	**21 (34.4)**	**26 (42.6)**	**13 (21.3)**
NHP	Human	72/82	87.8%	14 (19.4)	24 (33.3)	19 (26.4)	15 (20.1)
	NHP	76/86	88.4%	19 (25.0)	37 (48.7)	19 (25.0)	1 (1.3)

^1^NHP samples tested using human reagents shown in Bold. %CV criteria met with 6 dilutions indicates parallelism over a 32 fold range, 5 dilutions a 16 fold range, 4 dilutions and 8 fold range, 3 dilutions a 4 fold range, which is the minimum acceptable range for passing TS.

**Fig 1:**
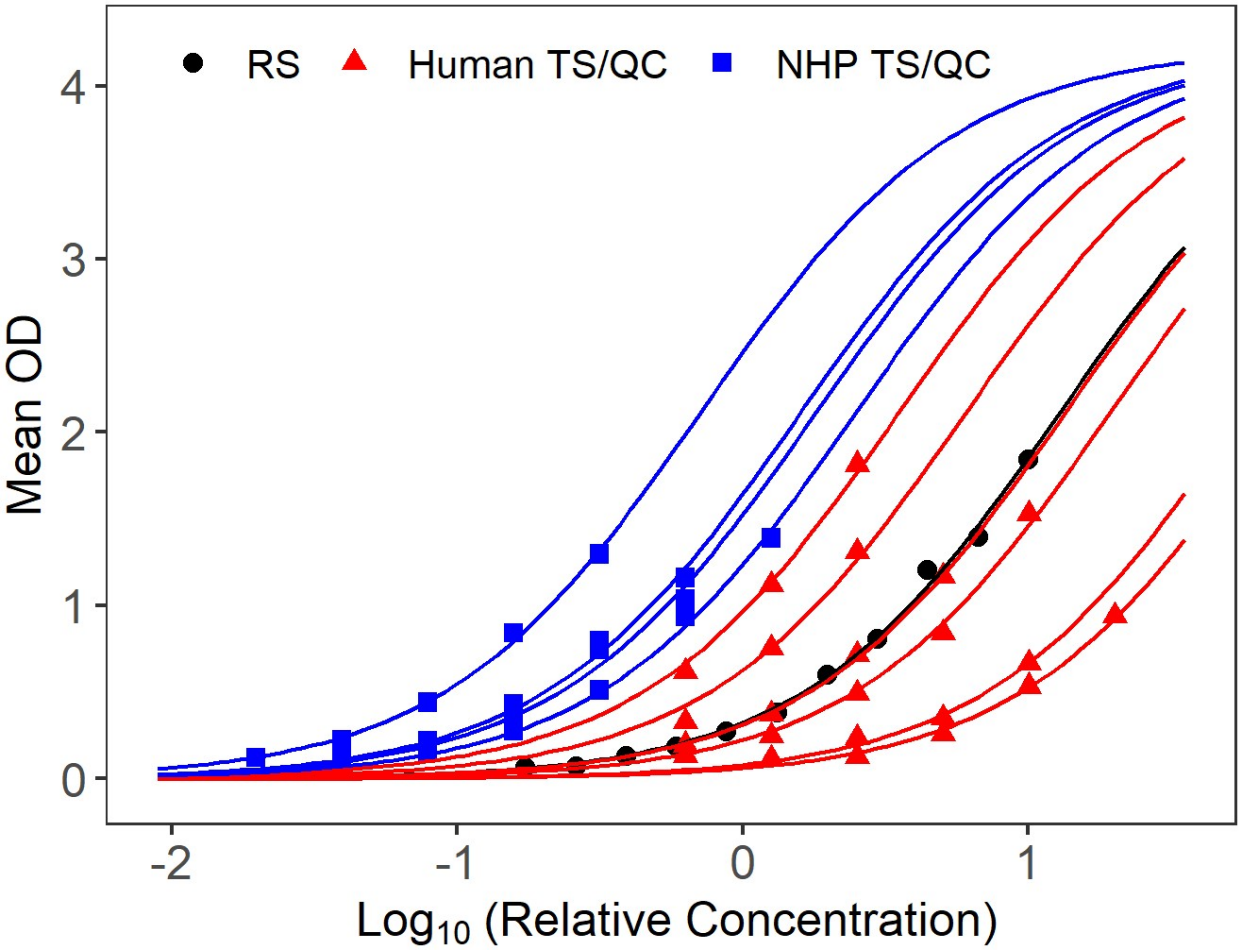
Sample of fitted modified 4PL model for a single plate with human RS/conjugate. RS displayed in black, NHP TS/QC in blue, human TS/QC in red.

**Fig 2:**
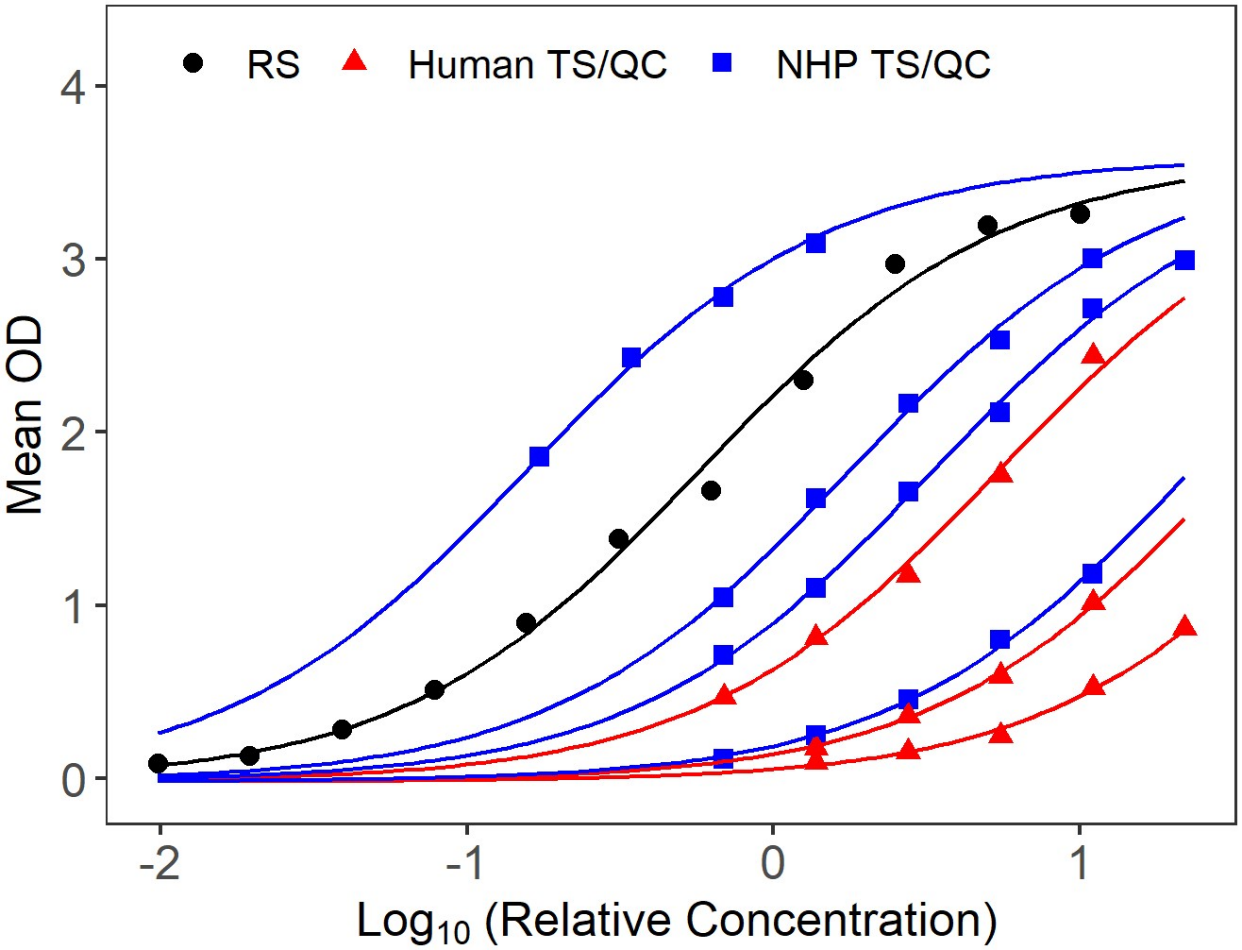
Sample of fitted modified 4PL model for a single plate with NHP RS/conjugate. RS displayed in black, NHP TS/QC in blue, human TS/QC in red.

**Fig 3:**
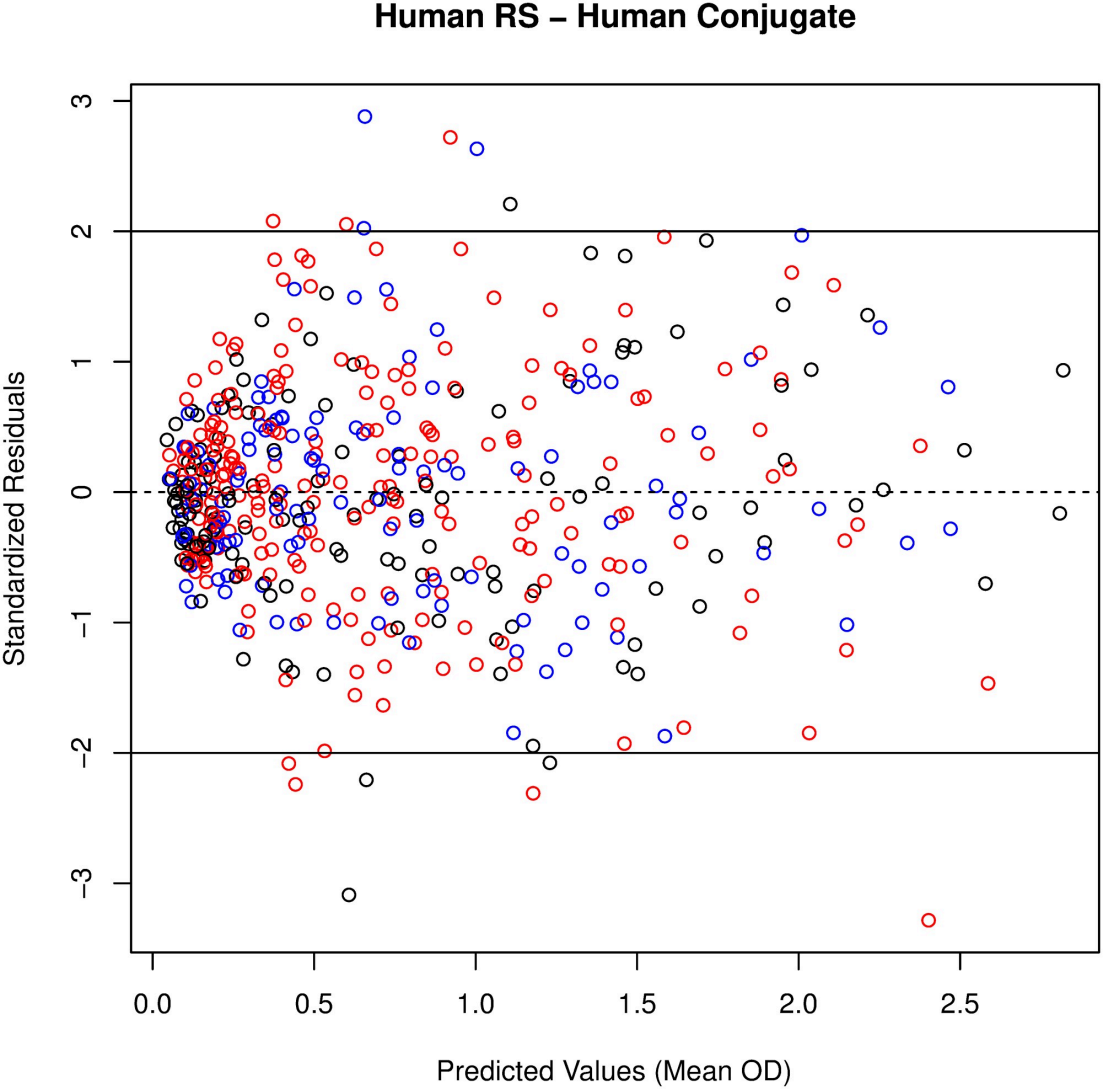
No pattern to studentized residuals plotted against predicted values from the modified 4PL model for all plates with human RS/conjugate indicates good fit. RS displayed in black, NHP TS/QC in blue, human TS/QC in red.

**Fig 4:**
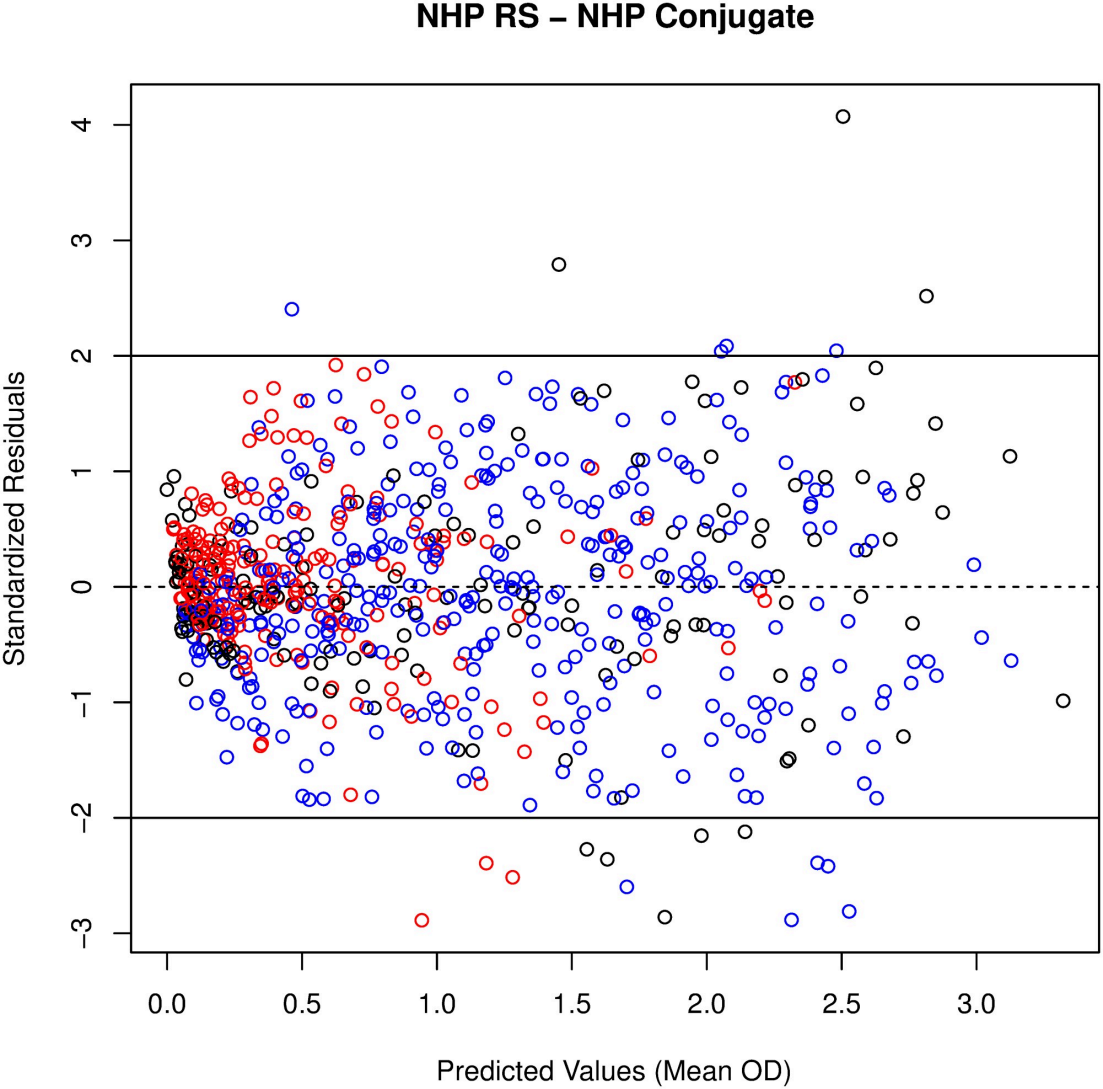
No pattern to studentized residuals plotted against predicted values from the modified 4PL model for all plates with NHP RS/conjugate indicates good fit. RS displayed in black, NHP TS/QC in blue, human TS/QC in red.

In addition to the Plikaytis method evaluation, a modified 4PL model with sample-dependent EC_50_ was fitted to the RS, TS, and QC data for each plate, to evaluate parallelism in a model framework. The 4PL models fit well for human and NHP TS, when tested against human (Fig 1) or NHP RS/conjugate (Fig 2). Because only six dilutions are used for TS and starting dilutions were not optimized to fit 4PL models, separate 4PL models could not be fit to the TS data. Therefore, Studentized residuals were used to assess parallelism between NHP TS, human TS, and NHP or human RS/conjugate. QC samples were included in the assessment as additional TS for each species. The residual plots of all modeled plates combined, show consistent, cloud-like patterns with no trend across the human and NHP samples and RS, when tested with human RS/conjugate (Fig 3) and NHP RS/conjugate (Fig 4). This pattern in the Studentized residuals is consistent with the model assumptions, including parallelism.

### NHP matrix qualification experiment

Following the method feasibility experiments that established the cross-species reactivity and parallelism between NHP TS and human RS/conjugate, assay performance was characterized through matrix qualification for NHP serum. The format for the anti-EBOV GP IgG ELISA using human RS/conjugate with human NC and human QC samples, which was previously validated for testing human serum samples, was used and system suitability criteria for the validated assay were applied [38]. Background levels of naïve NHP sera were used to determine the minimum starting dilution for a test sample and to calculate a 95% prediction interval for limit of background evaluation. The ELISA limit of detection (LOD), limits of quantification, repeatability, intermediate precision, and dilutional linearity were calculated from results obtained from 60 qualification test samples (QTS) generated from 5 parent samples obtained from NHPs previously determined to have high antibody levels based on testing conducted using species-specific RS/conjugate. Preliminary testing of these samples indicated a range of 3,500 to 35,000 ELISA units/mL when tested against the human RS. The parent samples were diluted in naïve NHP sera (Lot Number BMI300) to prepare five dilutional linearity dilution sets (ten dilution samples per dilution set). Thus, 50 unique samples were prepared. However, some were tested at multiple starting dilutions, thus a total of 60 Qualification Test Samples (QTS) were prepared. The QTSs were prepared once and shared among the test operators. A summary of the qualification results is presented in Table 3. These qualification results were used to inform the selection of appropriate validation acceptance criteria for the partial validation of the anti-GP IgG ELISA for analysis of NHP serum.

**Table 3:** Summary of anti-EBOV GP IgG ELISA qualification results for NHP samples.

Parameter	Result
Negative Sample OD	95% prediction interval of 0.000 to 0.250
Limit of Detection	52.85 ELISA units/mL
Lower Limit of Quantification	54.87 ELISA units/mL
Upper Limit of Quantification	30,632.33 ELISA units/mL
Residual (Repeatability)	15.3% CV
Sum of Operator, Day Plate, Replicate Effects (Intermediate Precision)	19.9% CV
Total Assay Variability Due to Method (Intermediate Precision and Repeatability)	25.3% CV
Dilutional Linearity (Accuracy)	Slope of -0.99 with 90% confidence of (-1.02, -0.97)
Selectivity (Matrix Effects)	Percent difference (-5.1%, 13.2%).
Specificity	Mean ratio of Mock to 25 μg/mL rGP = 5.05
	Mean ratio of Mock to 10 μg/mL rGP = 4.06
	Mean ratio of Mock to 25 μg/mL CMV = 1.06

To generate a cutoff value for antibody-negative samples in the ELISA, a mixed-effects ANOVA model was fitted to the average OD values from 150 naïve NHP serum samples tested in the ELISA at starting dilutions of 1:25, 1:50, and 1:100. The mean OD for the 1:100 dilution was significantly less than the other dilutions, which were not significantly different from each other. Because there was no significant difference in the mean OD values of the 1:25 and 1:50 dilutions and to be consistent with the minimum starting dilution for human serum tested, the starting dilution of 1:50 was selected for further analysis. A 95% prediction interval for the OD of a new sample of naïve sera with a starting dilution of 1:50 was estimated as 0.000 to 0.250 OD; the upper bound of this interval (0.250) serves as the limit of background to determine negative test samples.

A logistic regression model estimating the probability of detection from the predicted sample concentration was used to determine the LOD as 52.85 ELISA units/mL. The LLOQ and ULOQ for the assay are 54.87 and 30,632.33 ELISA units/mL, respectively. This was a conservative estimate for the ULOQ based on the NHP samples available for testing in the qualification.

The random coefficients regression model relating log_10_(concentration) to log_10_(spike level) fit to data within the assay range (54.87 to 30,632.33 ELISA units/mL) had a slope of -0.99 and the 90% confidence interval for the slope was (-1.02, -0.97). The slopes were also estimated separately from each test specimen. The slopes ranged from -1.01 to -0.97, providing further evidence of dilutional linearity across all test specimens.

Total assay variability due to method is defined as the sum of intermediate precision and repeatability. Assay precision was evaluated using results from the 60 QTSs using data within the assay range (54.87 to 30,632.33 ELISA units/mL) as percent coefficient of variation (%CV) for repeatability (15.3%), intermediate precision (19.9%), and total assay variability (25.3%).

The selectivity of the assay, or the effect of sample matrix on assay performance and detection of antibodies, was evaluated by spiking one positive NHP serum sample (NHP RS, BMIZAIRE007) and one positive human serum sample (Human RS, BMIZAIRE102) into six naïve samples (NHP Negative Control BMI300, four individual naïve NHP serum samples, and ELISA Diluent) at six spike levels. The mean percent difference between the observed and expected concentration across the five naïve samples for each combination of positive test sample and spike level ranged from -25.0 to 36.0%. The 90% confidence interval for % difference calculated over all samples was (-5.1%, 13.2%).

The purpose of evaluating specificity was to assess the ability of the analytical method to differentiate the analyte from nonspecific analytes that may also be present in the sample matrix. Specificity was evaluated using five positive NHP serum samples and one naïve NHP serum sample that were pre-adsorbed with EBOV Kikwit rGP antigen (at 25 μg/mL and 10 μg/mL), CMV (Cytomegalovirus) antigen (at 25 μg/mL), or ELISA Diluent (mock). The mean ratio of mock (ELISA Diluent) to 25 μg/mL rGP concentration across the test samples was 5.05 with a lower 90% confidence bound of 3.61. The mean ratio of mock concentration to 10 μg/mL GP concentration across the test samples was 4.06 with a lower 90% confidence bound of 3.26. The mean ratio of mock concentration to 25 μg/mL CMV concentration across the test samples was 1.06 with an upper 90% confidence bound of 1.15. These results are consistent with the assay specifically measuring anti-GP IgG antibody concentrations.

### NHP partial validation

The anti-EBOV GP IgG ELISA validation addressed the limit of detection, limits of quantification, relative accuracy via dilutional linearity, precision (intermediate precision, repeatability, total assay variability), and selectivity for both concentration (ELISA units/mL) and endpoint titer responses. Table 4 provides the validation parameters, acceptance criteria, and results for the detection of EBOV GP-specific IgG in NHP serum samples. The acceptance criteria were set based on the results obtained during qualification of the assay for NHP serum samples and are consistent with the acceptance criteria applied to the human ELISA validation.

**Table 4:** Summary of anti-EBOV GP IgG ELISA partial validation parameters, acceptance criteria, and results for NHP samples.

Validation Parameter	Validation Acceptance Criteria	Validation Result	Pass/Fail Status
Limit of Detection (LOD)	Concentration ≤75 ELISA units/mL	Concentration LOD = 28.817 ELISA units/mL	Pass
	Endpoint titer ≤200	Endpoint titer LOD = 50	Pass
Lower Limit of Quantitation (LLOQ)	Concentration ≤75 ELISA units/mL	Concentration LLOQ = 31.076 ELISA units/mL	Pass
	Endpoint titer ≤200	Endpoint titer LLOQ = 50	Pass
Upper Limit of Quantitation (ULOQ)	NA—Presented for informational purposes only	Concentration ULOQ = 193,234.006 ELISA units/mL	NA
		Endpoint titer ULOQ = 204,800	NA
**Dilutional Linearity**			
Slope (Spike Level)	Concentration: (-1.20, -0.80)	Concentration: (-1.01, -1.00)	Pass
	Endpoint titer: (-1.20, -0.80)	Endpoint titer: (-1.02, -0.98)	Pass
Slope (Starting Dilution)	Concentration: (-0.20, 0.20)	Concentration: (0.04, 0.09)	Pass
	Endpoint titer: (-0.20, 0.20)	Endpoint titer: (-0.01, 0.02)	Pass
**Precision**			
Repeatability	Concentration: %CV < 20%	Concentration: 16.1% CV	Pass
	Endpoint titers: ≥ 80% with ≤10% of endpoint titers outside of one two-fold difference from the median upon repeat testing (repeatability)	Endpoint titer: 100% of the replicates on each plate where repeatability was tested were within one two-fold dilution of their respective median.	Pass
Total Assay Variability	Concentration: %CV < 35%	Concentration: 21.2% CV	Pass
	Endpoint titers: ≥ 80% with ≤10% of endpoint titers outside of one two-fold difference from the median titer calculated from plates across days and operators	Endpoint titer: 98% of VTSs had no more than 10% of endpoint titers outside one two-fold dilution of the median across all plates/days/operators	Pass
Selectivity (Matrix Effects)	Concentration: -35% TO 35%	Concentration: (-7.8%, 4.9%)	Pass
	Endpoint titers: 0.34 t0 3.00	Endpoint titer: (0.95, 1.13)	Pass

Using logistic regression analysis to predict the probability that a concentration or endpoint titer could be determined as a function of its predicted concentration or endpoint titer, an LOD was calculated for both concentration (28.817 ELISA units/mL) and endpoint titer (50). Both passed the validation acceptance criteria, which was a concentration no greater than 75 ELISA units/mL for concentration and an endpoint titer no greater than 100.The concentration LLOQ was estimated at 31.076 ELISA units/mL and the endpoint titer LLOQ was estimated at 50. Both passed the validation acceptance criteria, which was a concentration no greater than 75 ELISA units/mL and an endpoint titer no greater than 200. For informational purposes only, the ULOQ was also calculated and set at 193,234.006 ELISA units/mL for concentration and 204,800 for endpoint titer. These upper limits of quantitation are likely conservative estimates because of the limited availability of high concentration samples at the time of this validation.

For concentration with respect to spike level, the regression model relating log10(concentration) to log10(spike level) was fit to the data from all test samples excluding those values less than 31.076 ELISA units/mL (LLOQ) or greater than 193,234.006 ELISA units/mL (ULOQ). The slope was -1.01 and the 90% confidence interval for the slope was (-1.01, -1.00). Because the 90% confidence interval is entirely contained within the validation acceptance interval of (-1.20, -0.80), the assay passes with respect to dilutional linearity due to spike level. For concentration with respect to starting dilution, a regression model relating log10[concentration*spike] to log10 (starting dilution) was refit to the data from all test samples excluding those values less than 31.076 ELISA units/mL or greater than 193,234.006 ELISA units/mL. The slope was 0.06 and the 90% confidence interval for the slope was (0.04, 0.09). Because the 90% confidence interval is entirely contained within the validation acceptance interval of (-0.20, 0.20), the assay passes with respect to dilutional linearity due to starting dilution.

For endpoint titer with respect to spike level, the regression model relating log10(endpoint titer) to log10(spike level) was fit to the data from all test samples excluding those values less than 50 or greater than 204,800. The slope was -1.00 and the 90% confidence interval for the slope was (-1.02, -0.98). Because the 90% confidence interval is entirely contained within the validation acceptance interval of (-1.20, -0.80), the assay passes with respect to dilutional linearity due to spike level. For endpoint titer with respect to starting dilution, a regression model relating log10[endpoint titer*spike] to log10 (starting dilution) was refit to the data from all test samples excluding those values less than 50 or greater than 204,800. The slope was 0.00 and the 90% confidence interval for the slope was (-0.01, 0.02). Because the 90% confidence interval is entirely contained within the validation acceptance interval of (-0.20, 0.20), the assay passes with respect to dilutional linearity due to starting dilution.

For concentration, the repeatability percent CV was calculated to be 16.1%, which is less than the validation acceptable limit of 20%; and total assay variability percent CV was calculated to be 21.2%, which is less than the validation acceptable limit of 35%. Thus, the assay passes with respect to both parameters for concentration. Although there were no formal acceptance criteria for intermediate precision, it was estimated at 13.6%.

For endpoint titer, 98% of test samples had no more than 10% of endpoint titers outside of one two-fold difference from the median on all plates tested across days and operators. Because this percentage is greater than the acceptance criteria of 80%, the total assay variability is acceptable. All 31 test samples for which repeatability testing was performed had 100% of endpoint titers within one two-fold difference of the median. Because this percentage is greater than the acceptance criteria of 80%, the assay repeatability is acceptable.

Based upon the evaluation of the validation parameters, the working range of the assay is 31.076–193,234.006 ELISA units/mL with respect to concentration and 50–204,800 with respect to endpoint titer. It is within this range that the assay has acceptable accuracy, precision, and dilutional linearity. Note that the upper range of these limits (the ULOQ) was calculated based on the range of the samples available for testing in this validation. The true ULOQ is likely to be greater and may be re-evaluated if higher concentration samples become available.

For selectivity with respect to concentration, the percent difference of the observed concentration of three positive serum samples spiked at two concentrations into five individual naïve serum samples compared to the observed concentration of spikes into a comparator naïve serum sample (BMI300) was calculated. The mean percent difference across the five naive test samples and a corresponding 90% confidence interval on this mean were calculated. Likewise, a fold difference was calculated for evaluation of the endpoint titer. An equivalence analysis was conducted (e.g., two one-sided test) for each endpoint to determine if the confidence interval is completely contained within the validation acceptance interval of (-35%, 35%) for concentrations and (0.34, 3.00) for endpoint titer. For concentration, the confidence interval of (-7.8%, 4.9%) is completely contained within the validation acceptance interval of (-35%, 35%). For endpoint titer, the confidence interval of (0.95, 1.13) is completely contained within the validation acceptance interval of (0.34, 3.00). Thus, both concentration and endpoint titer pass with respect to selectivity.

## Discussion

This study provides test data to support the use of the EBOV anti-GP IgG ELISA for evaluation of NHP test samples.

Method feasibility experiments confirmed that the human conjugate concentration used in the human assay was acceptable for NHP samples. All conjugate dilutions tested (1:4,000 to 1:16,000) were found to be suitable in the assay thus efforts were focused on a dilution of 1:10,000.

The method feasibility study also evaluated parallelism between human and NHP RS and TS when samples were tested using human RS/conjugate and NHP RS/conjugate. NHP samples tested on plates using human RS/conjugate met the %CV acceptance criterion 85.9% of the time, compared to 95.1% of the time for human TS on those plates. Although the proportion passing was somewhat lower for NHP than human samples, NHP samples tested using human RS/conjugate passed at similar rates as NHP samples tested using NHP RS/conjugate. NHP and human TS met the %CV criterion with similar rates (88.4 and 87.8%, respectively) when tested against NHP RS/conjugate. For both test conditions, the majority of TS and QC samples met the %CV criterion over four to six dilutions, which indicates parallelism over an 8-fold to 32-fold range over the dilution series. The high proportions and similarity of success rates over these test conditions supports parallelism between TS/QC samples and the RS-conjugates across species. Residual analysis from modified 4PL models with a common slope, common upper and lower asymptotes, and sample dependent EC50 fit to the RS and all passing TS and QC samples from each plate provided additional support for parallelism between RS and TS/QC samples for the human RS/conjugate and NHP RS/conjugate test conditions.

Following the method feasibility experiments that established the cross-species reactivity and parallelism between NHP TS and human RS/conjugate, assay performance was characterized through qualification and robustness testing for NHP serum. The purpose of the qualification was to evaluate the performance characteristics of NHP serum tested in the human assay prior to performance of a partial validation that supports testing of NHP serum in the human assay. The qualification provides a thorough characterization of the performance of NHP samples tested in the human anti-EBOV IgG ELISA. The results were used as a foundation for guiding assay validation acceptance criteria and interpretation of results when testing NHP samples from non-clinical studies.

Finally, the anti-GP IgG ELISA using human RS and human conjugate was validated for analysis of NHP serum to quantify anti-GP IgG antibodies by investigating the following validation parameters: Precision (Repeatability and Total Assay Variability), Dilutional Linearity, LLOQ, LOD, and Selectivity. Two assay endpoints were evaluated: concentration (ELISA units/mL) and endpoint titer. Either or both endpoints may be used as the final assay readout depending on the intended purpose. However, because of discrete and discontinuous nature of the endpoint titer and because the concentration is a relative measure calculated based on the RS of the plate, the concentration endpoint is a more precise measure and will have overall better utility than the endpoint titer. Thus, concentration is the recommended assay readout. The validation criteria were satisfied for all validation parameters evaluated, for both assay endpoints. Based upon these results, the anti-EBOV GP IgG ELISA method has been validated for use with NHP serum samples.

## Conclusions

It is advantageous for bridging studies conducted in support of licensure under the FDA Animal Rule that the immune response in human and NHP samples be tested using the same reagents and assay. These studies provide data to confirm the adequacy of the EBOV anti-GP IgG ELISA for testing both human and NHP serum samples. Qualification and validation experiments were performed to characterize the anti-EBOV GP IgG ELISA (previously validated for human sample testing) for use with NHP TS. The validation criteria were satisfied for all validation parameters evaluated, for both concentration and endpoint titer responses. Based upon these results, the anti-EBOV GP IgG ELISA method has been validated for use with NHP serum samples. In the future, NHP samples from non-clinical studies or other studies may be tested using this validated assay to determine the presence of EBOV anti-GP IgG antibodies generated in response either to inoculation with an Ebola virus vaccine or from exposure to Ebola virus.

## Supporting information

S1 Fig Method feasibility experiment NHP reference standard dilution and plate layout for cross-species conjugate concentration. (TIF)

S2 Fig Method feasibility experiment human conjugate dilution and plate layout for cross-species conjugate concentration. (TIF)

S3 Fig Method feasibility experiment modified plate layout for testing human and NHP samples. (TIF)

S1 Table Human and NHP test samples used for method feasibility study. (DOCX)

S2 Table Concentrations and target starting dilutions of NHP samples tested using human RS and QC in the anti-EBOV GP IgG ELISA in preliminary testing. (DOCX)

S3 Table Preparation of dilutional linearity qualification test samples. (DOCX)

S4 Table Preparation of matrix effects qualification test samples. (DOCX)

S5 Table Preparation of specificity qualification test samples. (DOCX)

S6 Table Preparation of validation test samples. (DOCX)

S7 Table Plate layout and optical density results used to determine conjugate dilution. (DOCX)
