# Supplementary figures and images for: Method feasibility for cross-species testing, qualification, and validation of the Filovirus Animal Nonclinical Group anti-Ebola virus glycoprotein immunoglobulin G enzyme-linked immunosorbent assay for non-human primate serum samples
Source: PLoS One. 2020 Oct 29;15(10):e0241016. doi: 10.1371/journal.pone.0241016 (PMC7595334; doi:10.1371/journal.pone.0241016)

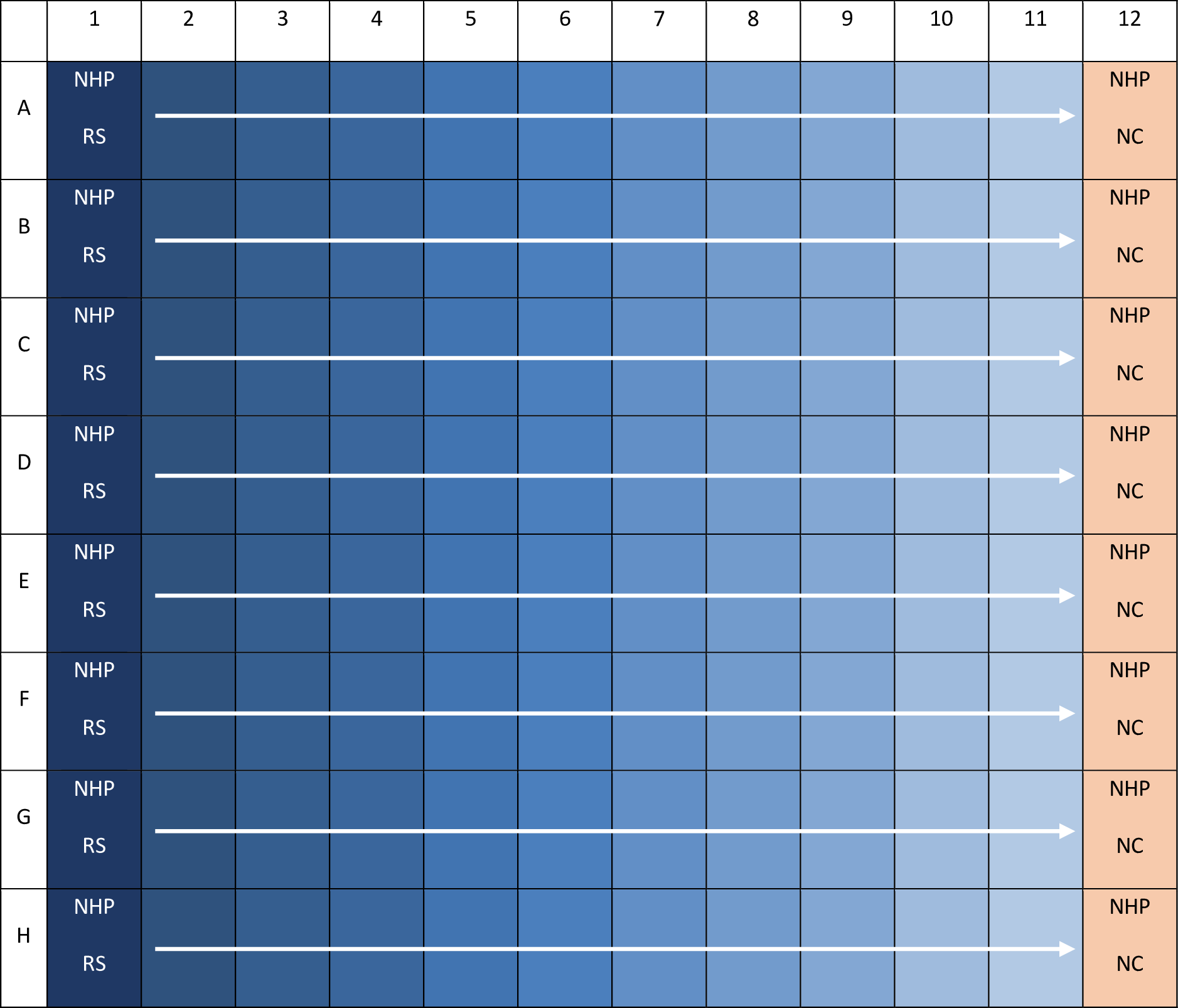

Supplement: S1 Fig — (TIF) [file pone.0241016.s001.tif]

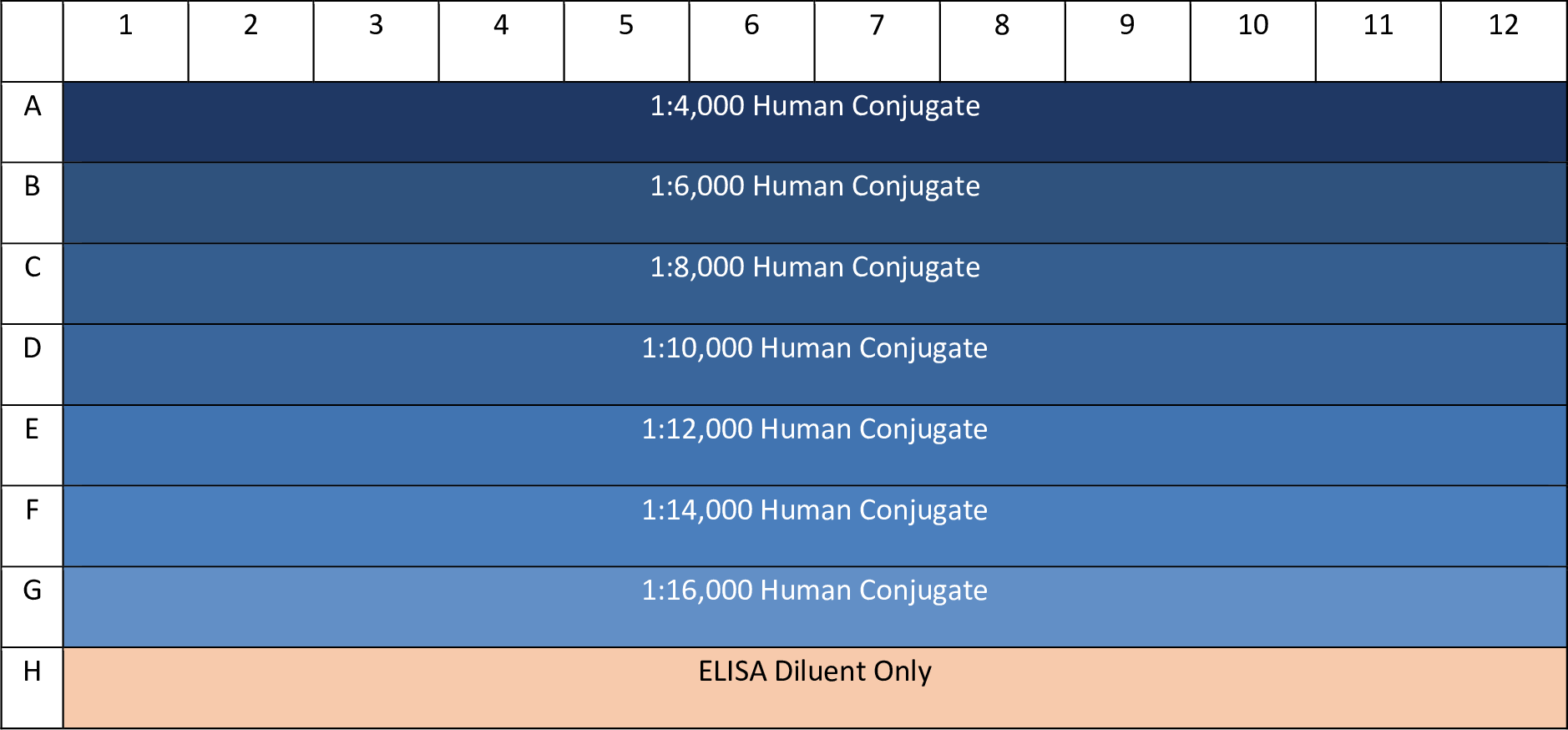

Supplement: S2 Fig — (TIF) [file pone.0241016.s002.tif]

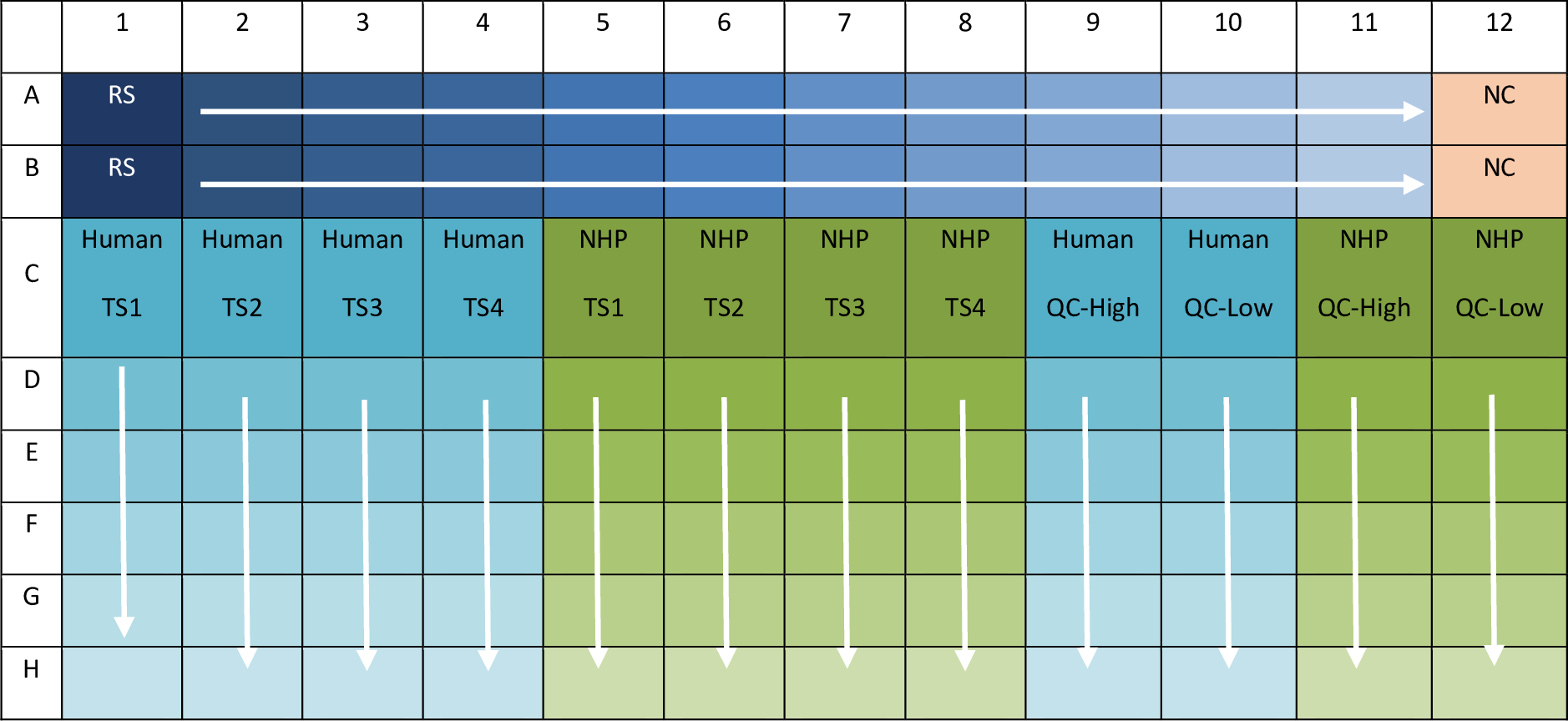

Supplement: S3 Fig — (TIF) [file pone.0241016.s003.tif]
