# Supplementary material for: Method feasibility for cross-species testing, qualification, and validation of the Filovirus Animal Nonclinical Group anti-Ebola virus glycoprotein immunoglobulin G enzyme-linked immunosorbent assay for non-human primate serum samples
Source: PLoS One. 2020 Oct 29;15(10):e0241016. doi: 10.1371/journal.pone.0241016 (PMC7595334; doi:10.1371/journal.pone.0241016)
Supplement: S1 Table — (DOCX) [file pone.0241016.s004.docx]

**S1 Table.** **Human and NHP Test Samples used for Method Feasibility Study.**

| **TS Number** | **Test Group** | **Anticipated Concentration (ELISA Units/mL)^1^** |
| --- | --- | --- |
| **Human TS1** | 1 | 599.32 |
| **Human TS2** | 1 | 3453.56 |
| **Human TS3** | 1 | 1082.08 |
| **Human TS4** | 1 | 461.68 |
| **Human TS5** | 2 | 776.88 |
| **Human TS6** | 2 | 2399.55 |
| **Human TS7** | 2 | 176.94 |
| **Human TS8** | 2 | 990.11 |
| **Human TS9** | 3 | 626.69 |
| **Human TS10** | 3 | 2605.82 |
| **Human TS11** | 3 | 222.59 |
| **Human TS12** | 3 | 769.59 |
| **NHP TS1** | 1 | 1374.49 |
| **NHP TS2** | 1 | 753.23 |
| **NHP TS3** | 1 | 374.29 |
| **NHP TS4** | 1 | 3468.03 |
| **NHP TS5** | 2 | 924.84 |
| **NHP TS6** | 2 | 1775.66 |
| **NHP TS7** | 2 | 1045.02 |
| **NHP TS8** | 2 | 655.63 |
| **NHP TS9** | 3 | 192.67 |
| **NHP TS10** | 3 | 1008.79 |
| **NHP TS11** | 3 | 2530.82 |
| **NHP TS12** | 3 | 859.90 |
