# Supplementary material for: Method feasibility for cross-species testing, qualification, and validation of the Filovirus Animal Nonclinical Group anti-Ebola virus glycoprotein immunoglobulin G enzyme-linked immunosorbent assay for non-human primate serum samples
Source: PLoS One. 2020 Oct 29;15(10):e0241016. doi: 10.1371/journal.pone.0241016 (PMC7595334; doi:10.1371/journal.pone.0241016)
Supplement: S2 Table — (DOCX) [file pone.0241016.s005.docx]

**S2 Table.** **Concentrations and Target Starting Dilutions of NHP Samples Tested using Human RS and QC in the Anti-EBOV GP IgG ELISA in Preliminary Testing**

| **Test Sample** | **Starting Dilution Used for Pre-testing** | **Average ELISA Units/mL** | **Standard Deviation** | **%CV** | **Target Starting Dilution** |
| --- | --- | --- | --- | --- | --- |
| BMIZAIRE010* | 1600 | 7756.11 | 1979.05 | 25.52% | 800 |
| BMIZAIRE007* | 1600 | 8607.39 | 1478.22 | 17.17% | 800 |
| BMIZAIRE004* | 800 | 3651.15 | 1032.41 | 28.28% | 400 |
| 05400.09984.D59* | 6400 | 34,844.28 | 9910.70 | 28.44% | 3200 |
| 05400.12633.D59* | 3200 | 14,461.58 | 2243.09 | 15.51% | 1600 |
| 05400.00412.D59 | 3200 | 13,803.81 | 3135.14 | 22.71% | 1600 |
| 05400.05756.D59 | 2000 | 12,852.00 | 2852.12 | 22.19% | 1600 |
| 05400.07511.D59 | 3200 | 18,056.54 | 4677.79 | 25.91% | 1600 |
| BMI300 | 50 | 0.00 | N/A | N/A | 50 |
| CYN179176* | 50 | 0.00 | N/A | N/A | 50 |
| CYN179177* | 50 | 0.00 | N/A | N/A | 50 |
| CYN179178 | 50 | 0.00 | N/A | N/A | 50 |
| CYN179179 | 50 | 0.00 | N/A | N/A | 50 |
| CYN179180 | 50 | 0.00 | N/A | N/A | 50 |
| CYN179181 | 50 | 0.00 | N/A | N/A | 50 |
| CYN179182 | 50 | 0.00 | N/A | N/A | 50 |
| CYN179183 | 50 | 0.00 | N/A | N/A | 50 |
| CYN179184* | 50 | 0.00 | N/A | N/A | 50 |
| CYN179185* | 50 | 0.00 | N/A | N/A | 50 |

*TS selected for qualification study.
