# Supplementary material for: Method feasibility for cross-species testing, qualification, and validation of the Filovirus Animal Nonclinical Group anti-Ebola virus glycoprotein immunoglobulin G enzyme-linked immunosorbent assay for non-human primate serum samples
Source: PLoS One. 2020 Oct 29;15(10):e0241016. doi: 10.1371/journal.pone.0241016 (PMC7595334; doi:10.1371/journal.pone.0241016)
Supplement: S3 Table — (DOCX) [file pone.0241016.s006.docx]

**S3 Table.** **Preparation of Dilutional Linearity Qualification Test Samples**

| **QTS #** | **Test Specimen ID** | **Anti-GP IgG Concentration  (ELISA Units/mL) (Before Spike)^1^** | **Final Dilution Factor (Spike)** | **Negative Specimen Identifier (Diluent)** | **Expected QTS Anti-GP IgG Concentration  (ELISA Units/mL)^1^** | **QTS Starting Dilution** |
| --- | --- | --- | --- | --- | --- | --- |
| 1 | BMIZAIRE010 | 1033 | 1:1 | NA | 1033 | 1:800 |
| 2 | Same as QTS#1 | 1033 | 1:1 | NA | 1033 | 1:1600 |
| 3 | Same as QTS#1 | 1033 | 1:1 | NA | 1033 | 1:400 |
| 4 | BMIZAIRE010 | 1033 | 1:2 | BMI300 | 517 | 1:400 |
| 5 | BMIZAIRE010 | 1033 | 1:4 | BMI300 | 258 | 1:200 |
| 6 | BMIZAIRE010 | 1033 | 1:8 | BMI300 | 129 | 1:100 |
| 7 | BMIZAIRE010 | 1033 | 1:16 | BMI300 | 65 | 1:50 |
| 8 | BMIZAIRE010 | 129 | 1:32 | BMI300 | 32 | 1:50 |
| 9 | BMIZAIRE010 | 129 | 1:64 | BMI300 | 16 | 1:50 |
| 10 | BMIZAIRE010 | 65 | 1:128 | BMI300 | 8 | 1:50 |
| 11 | BMIZAIRE010 | 65 | 1:256 | BMI300 | 4 | 1:50 |
| 12 | BMIZAIRE010 | 65 | 1:512 | BMI300 | 2 | 1:50 |
| 13 | BMIZAIRE007 | 1104 | 1:1 | NA | 1104 | 1:800 |
| 14 | BMIZAIRE007 | 1104 | 1:2 | BMI300 | 552 | 1:400 |
| 15 | Same as QTS#14 | 1104 | 1:2 | BMI300 | 552 | 1:800 |
| 16 | Same as QTS#14 | 1104 | 1:2 | BMI300 | 552 | 1:200 |
| 17 | BMIZAIRE007 | 1104 | 1:4 | BMI300 | 276 | 1:200 |
| 18 | BMIZAIRE007 | 1104 | 1:8 | BMI300 | 138 | 1:100 |
| 19 | BMIZAIRE007 | 1104 | 1:16 | BMI300 | 69 | 1:50 |
| 20 | BMIZAIRE007 | 138 | 1:32 | BMI300 | 35 | 1:50 |
| 21 | BMIZAIRE007 | 138 | 1:64 | BMI300 | 17 | 1:50 |
| 22 | BMIZAIRE007 | 69 | 1:128 | BMI300 | 9 | 1:50 |
| 23 | BMIZAIRE007 | 69 | 1:256 | BMI300 | 4 | 1:50 |
| 24 | BMIZAIRE007 | 69 | 1:512 | BMI300 | 2 | 1:50 |
| 25 | BMIZAIRE004 | 453 | 1:1 | NA | 453 | 1:400 |
| 26 | Same as QTS#25 | 453 | 1:1 | NA | 453 | 1:800 |
| 27 | Same as QTS#25 | 453 | 1:1 | NA | 453 | 1:200 |
| 28 | BMIZAIRE004 | 453 | 1:2 | BMI300 | 227 | 1:200 |
| 29 | BMIZAIRE004 | 453 | 1:4 | BMI300 | 113 | 1:100 |
| 30 | BMIZAIRE004 | 453 | 1:8 | BMI300 | 57 | 1:50 |
| 31 | BMIZAIRE004 | 453 | 1:16 | BMI300 | 28 | 1:50 |
| 32 | BMIZAIRE004 | 57 | 1:32 | BMI300 | 14 | 1:50 |
| 33 | BMIZAIRE004 | 57 | 1:64 | BMI300 | 7 | 1:50 |

**S3 Table. Preparation of Dilutional Linearity Qualification Test Samples (continued)**

| **QTS #** | **Test Specimen ID** | **Anti-GP IgG Concentration  (ELISA Units/mL) (Before Spike)^1^** | **Final Dilution Factor (Spike)** | **Negative Specimen Identifier (Diluent)** | **Expected QTS Anti-GP IgG Concentration  (ELISA Units/mL)^1^** | **QTS Starting Dilution** |
| --- | --- | --- | --- | --- | --- | --- |
| 34 | BMIZAIRE004 | 28 | 1:128 | BMI300 | 4 | 1:50 |
| 35 | BMIZAIRE004 | 28 | 1:256 | BMI300 | 2 | 1:50 |
| 36 | BMIZAIRE004 | 28 | 1:512 | BMI300 | 1 | 1:50 |
| 37 | 05400.09984.D59 | 4744 | 1:1 | NA | 4744 | 1:3200 |
| 38 | 05400.09984.D59 | 4744 | 1:2 | BMI300 | 2372 | 1:1600 |
| 39 | 05400.09984.D59 | 4744 | 1:4 | BMI300 | 1186 | 1:800 |
| 40 | 05400.09984.D59 | 4744 | 1:8 | BMI300 | 593 | 1:400 |
| 41 | 05400.09984.D59 | 4744 | 1:16 | BMI300 | 297 | 1:200 |
| 42 | Same as QTS#41 | 4744 | 1:16 | BMI300 | 297 | 1:400 |
| 43 | Same as QTS#41 | 4744 | 1:16 | BMI300 | 297 | 1:100 |
| 44 | 05400.09984.D59 | 593 | 1:32 | BMI300 | 148 | 1:100 |
| 45 | 05400.09984.D59 | 593 | 1:64 | BMI300 | 74 | 1:50 |
| 46 | 05400.09984.D59 | 297 | 1:128 | BMI300 | 37 | 1:50 |
| 47 | 05400.09984.D59 | 297 | 1:256 | BMI300 | 19 | 1:50 |
| 48 | 05400.09984.D59 | 297 | 1:512 | BMI300 | 9 | 1:50 |
| 49 | 05400.12633.D59 | 2484 | 1:1 | NA | 2484 | 1:1600 |
| 50 | 05400.12633.D59 | 2484 | 1:2 | BMI300 | 1242 | 1:800 |
| 51 | 05400.12633.D59 | 2484 | 1:4 | BMI300 | 621 | 1:400 |
| 52 | 05400.12633.D59 | 2484 | 1:8 | BMI300 | 310 | 1:200 |
| 53 | 05400.12633.D59 | 2484 | 1:16 | BMI300 | 155 | 1:100 |
| 54 | Same as VTS#53 | 2484 | 1:16 | BMI300 | 155 | 1:200 |
| 55 | Same as VTS#53 | 2484 | 1:16 | BMI300 | 155 | 1:50 |
| 56 | 56 - Step 1 | 310 | 1:32 | BMI300 | 78 | 1:50 |
| 57 | 57 - Step 1 | 310 | 1:64 | BMI300 | 39 | 1:50 |
| 58 | 58 - Step 1 | 155 | 1:128 | BMI300 | 19 | 1:50 |
| 59 | 59 - Step 1 | 155 | 1:256 | BMI300 | 10 | 1:50 |
| 60 | 60 - Step 1 | 155 | 1:512 | BMI300 | 5 | 1:50 |

^1^Expected concentration based on results from the NHP anti-GP IgG ELISA. Results in the human anti-GP IgG ELISA are expected to be different (approximately 7-fold higher).

Alternating shading used to separate one QTS from the next. Dark shading used to indicate QTSs that are part of starting dilution evaluation.
