# Supplementary material for: Method feasibility for cross-species testing, qualification, and validation of the Filovirus Animal Nonclinical Group anti-Ebola virus glycoprotein immunoglobulin G enzyme-linked immunosorbent assay for non-human primate serum samples
Source: PLoS One. 2020 Oct 29;15(10):e0241016. doi: 10.1371/journal.pone.0241016 (PMC7595334; doi:10.1371/journal.pone.0241016)
Supplement: S4 Table — (DOCX) [file pone.0241016.s007.docx]

**S4 Table.** **Preparation of Matrix Effects Qualification Test Samples**

| **QTS #^1,2^** | **Test Specimen ID** | **Anti-GP IgG Concentration (ELISA Units/mL) (Before Spike)^1^** | **Final Dilution Factor (Spike)** | **Negative Specimen Identifier (Diluent)** | **Expected QTS Anti-GP IgG Concentration (ELISA Units/mL)^1^** | **QTS Starting Dilution** |
| --- | --- | --- | --- | --- | --- | --- |
| 61 | BMIZAIRE007 | 1104 | 1:1 | BMI300 | 1104 | 1:800 |
| 62 | BMIZAIRE007 | 1104 | 1:2 | BMI300 | 552 | 1:400 |
| 63 | BMIZAIRE007 | 1104 | 1:8 | BMI300 | 138 | 1:50 |
| 64 | BMIZAIRE007 | 1104 | 1:16 | BMI300 | 69 | 1:50 |
| 65 | BMIZAIRE007 | 138 | 1:64 | BMI300 | 17 | 1:50 |
| 66 | BMIZAIRE007 | 69 | 1:256 | BMI300 | 4 | 1:50 |
| 67 | BMIZAIRE007 | 1104 | 1:1 | CYN179176 | 1104 | 1:800 |
| 68 | BMIZAIRE007 | 1104 | 1:2 | CYN179176 | 552 | 1:400 |
| 69 | BMIZAIRE007 | 1104 | 1:8 | CYN179176 | 138 | 1:100 |
| 70 | BMIZAIRE007 | 1104 | 1:16 | CYN179176 | 69 | 1:50 |
| 71 | BMIZAIRE007 | 138 | 1:64 | CYN179176 | 17 | 1:50 |
| 72 | BMIZAIRE007 | 69 | 1:256 | CYN179176 | 4 | 1:50 |
| 73 | BMIZAIRE007 | 1104 | 1:1 | CYN179177 | 1104 | 1:800 |
| 74 | BMIZAIRE007 | 1104 | 1:2 | CYN179177 | 552 | 1:400 |
| 75 | BMIZAIRE007 | 1104 | 1:8 | CYN179177 | 138 | 1:50 |
| 76 | BMIZAIRE007 | 1104 | 1:16 | CYN179177 | 69 | 1:50 |
| 77 | BMIZAIRE007 | 138 | 1:64 | CYN179177 | 17 | 1:50 |
| 78 | BMIZAIRE007 | 69 | 1:256 | CYN179177 | 4 | 1:50 |
| 79 | BMIZAIRE007 | 1104 | 1:1 | CYN179184 | 1104 | 1:800 |
| 80 | BMIZAIRE007 | 1104 | 1:2 | CYN179184 | 552 | 1:400 |
| 81 | BMIZAIRE007 | 1104 | 1:8 | CYN179184 | 138 | 1:50 |
| 82 | BMIZAIRE007 | 1104 | 1:16 | CYN179184 | 69 | 1:50 |
| 83 | BMIZAIRE007 | 138 | 1:64 | CYN179184 | 17 | 1:50 |
| 84 | BMIZAIRE007 | 69 | 1:256 | CYN179184 | 4 | 1:50 |
| 85 | BMIZAIRE007 | 1104 | 1:1 | CYN179185 | 1104 | 1:800 |
| 86 | BMIZAIRE007 | 1104 | 1:2 | CYN179185 | 552 | 1:400 |
| 87 | BMIZAIRE007 | 1104 | 1:8 | CYN179185 | 138 | 1:50 |
| 88 | BMIZAIRE007 | 1104 | 1:16 | CYN179185 | 69 | 1:50 |
| 89 | BMIZAIRE007 | 138 | 1:64 | CYN179185 | 17 | 1:50 |
| 90 | BMIZAIRE007 | 69 | 1:256 | CYN179185 | 4 | 1:50 |
| 91 | BMIZAIRE007 | 1104 | 1:1 | ELISA Diluent | 1104 | 1:800 |
| 92 | BMIZAIRE007 | 1104 | 1:2 | ELISA Diluent | 552 | 1:400 |
| 93 | BMIZAIRE007 | 1104 | 1:8 | ELISA Diluent | 138 | 1:50 |
| 94 | BMIZAIRE007 | 1104 | 1:16 | ELISA Diluent | 69 | 1:50 |
| 95 | BMIZAIRE007 | 138 | 1:64 | ELISA Diluent | 17 | 1:50 |
| 96 | BMIZAIRE007 | 69 | 1:256 | ELISA Diluent | 4 | 1:50 |
| 97 | BMIZAIRE102 | 1009 | 1:1 | BMI300 | 1009 | 1:100 |
| 98 | BMIZAIRE102 | 1009 | 1:2 | BMI300 | 505 | 1:50 |
| 99 | BMIZAIRE102 | 1009 | 1:4 | BMI300 | 252 | 1:50 |
| 100 | BMIZAIRE102 | 1009 | 1:8 | BMI300 | 126 | 1:50 |
| 101 | BMIZAIRE102 | 1009 | 1:16 | BMI300 | 63 | 1:50 |

**S4 Table. Preparation of Matrix Effects Qualification Test Samples (continued)**

| **QTS #^1,2^** | **Test Specimen ID** | **Anti-GP IgG Concentration (ELISA Units/mL) (Before Spike)^1^** | **Final Dilution Factor (Spike)** | **Negative Specimen Identifier (Diluent)** | **Expected QTS Anti-GP IgG Concentration (ELISA Units/mL)^1^** | **QTS Starting Dilution** |
| --- | --- | --- | --- | --- | --- | --- |
| 102 | BMIZAIRE102 | 126 | 1:32 | BMI300 | 32 | 1:50 |
| 103 | BMIZAIRE102 | 1009 | 1:1 | CYN179176 | 1009 | 1:100 |
| 104 | BMIZAIRE102 | 1009 | 1:2 | CYN179176 | 505 | 1:50 |
| 105 | BMIZAIRE102 | 1009 | 1:4 | CYN179176 | 252 | 1:50 |
| 106 | BMIZAIRE102 | 1009 | 1:8 | CYN179176 | 126 | 1:50 |
| 107 | BMIZAIRE102 | 1009 | 1:16 | CYN179176 | 63 | 1:50 |
| 108 | BMIZAIRE102 | 126 | 1:32 | CYN179176 | 32 | 1:50 |
| 109 | BMIZAIRE102 | 1009 | 1:1 | CYN179177 | 1009 | 1:100 |
| 110 | BMIZAIRE102 | 1009 | 1:2 | CYN179177 | 505 | 1:50 |
| 111 | BMIZAIRE102 | 1009 | 1:4 | CYN179177 | 252 | 1:50 |
| 112 | BMIZAIRE102 | 1009 | 1:8 | CYN179177 | 126 | 1:50 |
| 113 | BMIZAIRE102 | 1009 | 1:16 | CYN179177 | 63 | 1:50 |
| 114 | BMIZAIRE102 | 126 | 1:32 | CYN179177 | 32 | 1:50 |
| 115 | BMIZAIRE102 | 1009 | 1:1 | CYN179184 | 1009 | 1:100 |
| 116 | BMIZAIRE102 | 1009 | 1:2 | CYN179184 | 505 | 1:50 |
| 117 | BMIZAIRE102 | 1009 | 1:4 | CYN179184 | 252 | 1:50 |
| 118 | BMIZAIRE102 | 1009 | 1:8 | CYN179184 | 126 | 1:50 |
| 119 | BMIZAIRE102 | 1009 | 1:16 | CYN179184 | 63 | 1:50 |
| 120 | BMIZAIRE102 | 126 | 1:32 | CYN179184 | 32 | 1:50 |
| 121 | BMIZAIRE102 | 1009 | 1:1 | CYN179185 | 1009 | 1:100 |
| 122 | BMIZAIRE102 | 1009 | 1:2 | CYN179185 | 505 | 1:50 |
| 123 | BMIZAIRE102 | 1009 | 1:4 | CYN179185 | 252 | 1:50 |
| 124 | BMIZAIRE102 | 1009 | 1:8 | CYN179185 | 126 | 1:50 |
| 125 | BMIZAIRE102 | 1009 | 1:16 | CYN179185 | 63 | 1:50 |
| 126 | BMIZAIRE102 | 126 | 1:32 | CYN179185 | 32 | 1:50 |
| 127 | BMIZAIRE102 | 1009 | 1:1 | ELISA Diluent | 1009 | 1:100 |
| 128 | BMIZAIRE102 | 1009 | 1:2 | ELISA Diluent | 505 | 1:50 |
| 129 | BMIZAIRE102 | 1009 | 1:4 | ELISA Diluent | 252 | 1:50 |
| 130 | BMIZAIRE102 | 1009 | 1:8 | ELISA Diluent | 126 | 1:50 |
| 131 | BMIZAIRE102 | 1009 | 1:16 | ELISA Diluent | 63 | 1:50 |
| 132 | BMIZAIRE102 | 126 | 1:32 | ELISA Diluent | 32 | 1:50 |

^1^This footnote only applied to BMIZAIRE007. Expected concentration based on results from the NHP anti-GP IgG ELISA. Results in the human anti-GP IgG ELISA are expected to be different (approximately 7-fold higher).

Alternating shading used to separate one QTS from the next.
