# Supplementary material for: Method feasibility for cross-species testing, qualification, and validation of the Filovirus Animal Nonclinical Group anti-Ebola virus glycoprotein immunoglobulin G enzyme-linked immunosorbent assay for non-human primate serum samples
Source: PLoS One. 2020 Oct 29;15(10):e0241016. doi: 10.1371/journal.pone.0241016 (PMC7595334; doi:10.1371/journal.pone.0241016)
Supplement: S5 Table — (DOCX) [file pone.0241016.s008.docx]

**S5 Table.** **Preparation of Specificity Qualification Test Samples**

| **QTS #** | **Test Specimen ID** | **Anti-GP IgG Concentration (ELISA Units/mL) (Before Spike)** | **Negative Specimen Identifier (Diluent)** | **Expected QTS Anti-GP IgG Concentration (ELISA Units/mL)^1^** | **QTS Starting Dilution** |
| --- | --- | --- | --- | --- | --- |
| 133 | BMIZAIRE010 | 1033 | Mock | 1033 | 1:800 |
| 134 | BMIZAIRE010 | 1033 | rGP – 10 µg/mL | 1033 | 1:800 |
| 135 | BMIZAIRE010 | 1033 | rGP - 25 µg/mL | 1033 | 1:800 |
| 136 | BMIZAIRE010 | 1033 | CMV - 25 µg/mL | 1033 | 1:800 |
| 137 | BMIZAIRE007 | 1104 | Mock | 1104 | 1:800 |
| 138 | BMIZAIRE007 | 1104 | rGP – 10 µg/mL | 1104 | 1:800 |
| 139 | BMIZAIRE007 | 1104 | rGP - 25 µg/mL | 1104 | 1:800 |
| 140 | BMIZAIRE007 | 1104 | CMV - 25 µg/mL | 1104 | 1:800 |
| 141 | 05400.00412.D59 | 1926 | Mock | 1926 | 1:1600 |
| 142 | 05400.00412.D59 | 1926 | rGP – 10 µg/mL | 1926 | 1:50 |
| 143 | 05400.00412.D59 | 1926 | rGP - 25 µg/mL | 1926 | 1:50 |
| 144 | 05400.00412.D59 | 1926 | CMV - 25 µg/mL | 1926 | 1:1600 |
| 145 | 05400.05756.D59 | 1571 | Mock | 1571 | 1:1600 |
| 146 | 05400.05756.D59 | 1571 | rGP – 10 µg/mL | 1571 | 1:50 |
| 147 | 05400.05756.D59 | 1571 | rGP - 25 µg/mL | 1571 | 1:50 |
| 148 | 05400.05756.D59 | 1571 | CMV - 25 µg/mL | 1571 | 1:1600 |
| 149 | 05400.07511.D59 | 2339 | Mock | 2339 | 1:1600 |
| 150 | 05400.07511.D59 | 2339 | rGP – 10 µg/mL | 2339 | 1:1600 |
| 151 | 05400.07511.D59 | 2339 | rGP - 25 µg/mL | 2339 | 1:1600 |
| 152 | 05400.07511.D59 | 2339 | CMV - 25 µg/mL | 2339 | 1:1600 |
| 153 | BMI300 | 0 | Mock | 0 | 1:50 |
| 154 | BMI300 | 0 | rGP – 10 µg/mL | 0 | 1:50 |
| 155 | BMI300 | 0 | rGP - 25 µg/mL | 0 | 1:50 |
| 156 | BMI300 | 0 | CMV - 25 µg/mL | 0 | 1:50 |

^1^ Expected concentration based on results from the NHP anti-GP IgG ELISA. Results in the human anti-GP IgG ELISA are expected to be different (approximately 7-fold higher).
