# Supplementary material for: Method feasibility for cross-species testing, qualification, and validation of the Filovirus Animal Nonclinical Group anti-Ebola virus glycoprotein immunoglobulin G enzyme-linked immunosorbent assay for non-human primate serum samples
Source: PLoS One. 2020 Oct 29;15(10):e0241016. doi: 10.1371/journal.pone.0241016 (PMC7595334; doi:10.1371/journal.pone.0241016)
Supplement: S6 Table — (DOCX) [file pone.0241016.s009.docx]

**S6 Table.** **Preparation of Validation Test Samples**

| **VTS #** | **Test Specimen ID** | **Anti-GP IgG Concentration  (ELISA Units/mL) (Before Spike)** | **Final Dilution Factor  (Spike)** | **Negative Specimen Identifier  (Diluent)** | **Expected VTS Anti-GP IgG Concentration  (ELISA Units/mL)** | **VTS Starting Dilution** |
| --- | --- | --- | --- | --- | --- | --- |
| 1 | 613.D56 (EBOV-2) | 233389 | 1:1 | NA | 233389 | 1:25600 |
| 2 | Same as VTS 1 | 233389 | 1:1 | NA | 233389 | 1:51200 |
| 3 | Same as VTS 1 | 233389 | 1:1 | NA | 233389 | 1:12800 |
| 4 | 613.D56 (EBOV-2) | 233389 | 1:5 | BMI300 | 46678 | 1:6400 |
| 5 | 613.D56 (EBOV-2) | 233389 | 1:25 | BMI300 | 9336 | 1:1600 |
| 6 | 613.D56 (EBOV-2) | 46678 | 1:125 | BMI300 | 1867 | 1:200 |
| 7 | 613.D56 (EBOV-2) | 9336 | 1:625 | BMI300 | 373 | 1:50 |
| 8 | 613.D56 (EBOV-2) | 1867 | 1:3125 | BMI300 | 75 | 1:50 |
| 9 | 613.D56 (EBOV-2) | 373 | 1:15625 | BMI300 | 15 | 1:50 |
| 10 | 613.D56 (EBOV-2) | 75 | 1:78125 | BMI300 | 3 | 1:50 |
| 11 | 242.49 (EBOV-2) | 203650 | 1:1 | NA | 203650 | 1:25600 |
| 12 | 242.49 (EBOV-2) | 203650 | 1:5 | BMI300 | 40730 | 1:6400 |
| 13 | Same as VTS 12 | 203650 | 1:5 | BMI300 | 40730 | 1:12800 |
| 14 | Same as VTS 12 | 203650 | 1:5 | BMI300 | 40730 | 1:3200 |
| 15 | 242.49 (EBOV-2) | 203650 | 1:25 | BMI300 | 8146 | 1:1600 |
| 16 | 242.49 (EBOV-2) | 40730 | 1:125 | BMI300 | 1629 | 1:200 |
| 17 | 242.49 (EBOV-2) | 8146 | 1:625 | BMI300 | 326 | 1:50 |
| 18 | 242.49 (EBOV-2) | 1629 | 1:3125 | BMI300 | 65 | 1:50 |
| 19 | 242.49 (EBOV-2) | 326 | 1:15625 | BMI300 | 13 | 1:50 |
| 20 | 242.49 (EBOV-2) | 65 | 1:78125 | BMI300 | 3 | 1:50 |
| 21 | 003-02.D49 (EBOV-2) | 187025 | 1:1 | NA | 187025 | 1:25600 |
| 22 | Same as VTS 21 | 187025 | 1:1 | NA | 187025 | 1:51200 |
| 23 | Same as VTS 21 | 187025 | 1:1 | NA | 187025 | 1:12800 |
| 24 | 003-02.D49 (EBOV-2) | 187025 | 1:5 | BMI300 | 37405 | 1:6400 |
| 25 | 003-02.D49 (EBOV-2) | 187025 | 1:25 | BMI300 | 7481 | 1:800 |
| 26 | 003-02.D49 (EBOV-2) | 37405 | 1:125 | BMI300 | 1496 | 1:200 |
| 27 | 003-02.D49 (EBOV-2) | 7481 | 1:625 | BMI300 | 299 | 1:50 |
| 28 | 003-02.D49 (EBOV-2) | 1496 | 1:3125 | BMI300 | 60 | 1:50 |
| 29 | 003-02.D49 (EBOV-2) | 299 | 1:15625 | BMI300 | 12 | 1:50 |
| 30 | 003-02.D49 (EBOV-2) | 60 | 1:78125 | BMI300 | 2 | 1:50 |

**S6 Table. Preparation of Validation Test Samples (continued)**

| **VTS #** | **Test Specimen ID** | **Anti-GP IgG Concentration  (ELISA Units/mL) (Before Spike)** | **Final Dilution Factor  (Spike)** | **Negative Specimen Identifier  (Diluent)** | **Expected VTS Anti-GP IgG Concentration  (ELISA Units/mL)** | **VTS Starting Dilution** |
| --- | --- | --- | --- | --- | --- | --- |
| 31 | M05748.D49 (EBOV-2) | 153253 | 1:1 | NA | 153253 | 1:25600 |
| 32 | Same as VTS 31 | 153253 | 1:1 | NA | 153253 | 1:51200 |
| 33 | Same as VTS 31 | 153253 | 1:1 | NA | 153253 | 1:12800 |
| 34 | M05748.D49 (EBOV-2) | 153253 | 1:5 | BMI300 | 30651 | 1:3200 |
| 35 | M05748.D49 (EBOV-2) | 153253 | 1:25 | BMI300 | 6130 | 1:800 |
| 36 | M05748.D49 (EBOV-2) | 30651 | 1:125 | BMI300 | 1226 | 1:200 |
| 37 | M05748.D49 (EBOV-2) | 6130 | 1:625 | BMI300 | 245 | 1:50 |
| 38 | M05748.D49 (EBOV-2) | 1226 | 1:3125 | BMI300 | 49 | 1:50 |
| 39 | M05748.D49 (EBOV-2) | 245 | 1:15625 | BMI300 | 10 | 1:50 |
| 40 | M05748.D49 (EBOV-2) | 49 | 1:78125 | BMI300 | 2 | 1:50 |
| 41 | 166.D49 (EBOV-2) | 80973 | 1:1 | NA | 80973 | 1:12800 |
| 42 | 166.D49 (EBOV-2) | 80973 | 1:5 | BMI300 | 16195 | 1:3200 |
| 43 | Same as VTS 42 | 80973 | 1:5 | BMI300 | 16195 | 1:6400 |
| 44 | Same as VTS 42 | 80973 | 1:5 | BMI300 | 16195 | 1:1600 |
| 45 | 166.D49 (EBOV-2) | 80973 | 1:25 | BMI300 | 3239 | 1:400 |
| 46 | 166.D49 (EBOV-2) | 16195 | 1:125 | BMI300 | 648 | 1:100 |
| 47 | 166.D49 (EBOV-2) | 3239 | 1:625 | BMI300 | 130 | 1:50 |
| 48 | 166.D49 (EBOV-2) | 648 | 1:3125 | BMI300 | 26 | 1:50 |
| 49 | 166.D49 (EBOV-2) | 130 | 1:15625 | BMI300 | 5 | 1:50 |
| 50 | 166.D49 (EBOV-2) | 26 | 1:78125 | BMI300 | 1 | 1:50 |
| 51 | 166.D63 (EBOV-2) | 57019 | 1:1 | NA | 57019 | 1:6400 |
| 52 | 166.D63 (EBOV-2) | 57019 | 1:5 | BMI300 | 11404 | 1:1600 |
| 53 | Same as VTS 52 | 57019 | 1:5 | BMI300 | 11404 | 1:3200 |
| 54 | Same as VTS 52 | 57019 | 1:5 | BMI300 | 11404 | 1:800 |
| 55 | 166.D63 (EBOV-2) | 57019 | 1:25 | BMI300 | 2281 | 1:400 |
| 56 | 56 - Step 1 | 11404 | 1:125 | BMI300 | 456 | 1:50 |
| 57 | 57 - Step 1 | 2281 | 1:625 | BMI300 | 91 | 1:50 |
| 58 | 58 - Step 2 | 1140 | 1:1250 | BMI300 | 46 | 1:50 |
| 59 | 59 - Step 2 | 570 | 1:2500 | BMI300 | 23 | 1:50 |
| 60 | 60 - Step 2 | 285 | 1:5000 | BMI300 | 11 | 1:50 |

**S6 Table. Preparation of Validation Test Samples (continued)**

| **VTS #** | **Test Specimen ID** | **Anti-GP IgG Concentration  (ELISA Units/mL) (Before Spike)** | **Final Dilution Factor  (Spike)** | **Negative Specimen Identifier  (Diluent)** | **Expected VTS Anti-GP IgG Concentration  (ELISA Units/mL)** | **VTS Starting Dilution** |
| --- | --- | --- | --- | --- | --- | --- |
| 61 | 577.D49 (EBOV-2) | 30774 | 1:1 | NA | 30774 | 1:3200 |
| 62 | Same as VTS 61 | 30774 | 1:1 | NA | 30774 | 1:6400 |
| 63 | Same as VTS 61 | 30774 | 1:1 | NA | 30774 | 1:1600 |
| 64 | 577.D49 (EBOV-2) | 30774 | 1:5 | BMI300 | 6155 | 1:800 |
| 65 | 577.D49 (EBOV-2) | 30774 | 1:25 | BMI300 | 1231 | 1:200 |
| 66 | 577.D49 (EBOV-2) | 6155 | 1:125 | BMI300 | 246 | 1:50 |
| 67 | 577.D49 (EBOV-2) | 1231 | 1:625 | BMI300 | 49 | 1:50 |
| 68 | 577.D49 (EBOV-2) | 615 | 1:1250 | BMI300 | 25 | 1:50 |
| 69 | 577.D49 (EBOV-2) | 308 | 1:2500 | BMI300 | 12 | 1:50 |
| 70 | 577.D49 (EBOV-2) | 154 | 1:5000 | BMI300 | 6 | 1:50 |
| 71 | 42.D168 (EBOV-1) | 11119 | 1:1 | NA | 11119 | 1:1600 |
| 72 | 42.D168 (EBOV-1) | 11119 | 1:5 | BMI300 | 2224 | 1:400 |
| 73 | Same as VTS 72 | 11119 | 1:5 | BMI300 | 2224 | 1:800 |
| 74 | Same as VTS 72 | 11119 | 1:5 | BMI300 | 2224 | 1:200 |
| 75 | 42.D168 (EBOV-1) | 11119 | 1:25 | BMI300 | 445 | 1:50 |
| 76 | 42.D168 (EBOV-1) | 2224 | 1:125 | BMI300 | 89 | 1:50 |
| 77 | 42.D168 (EBOV-1) | 445 | 1:625 | BMI300 | 18 | 1:50 |
| 78 | 42.D168 (EBOV-1) | 222 | 1:1250 | BMI300 | 9 | 1:50 |
| 79 | 42.D168 (EBOV-1) | 111 | 1:2500 | BMI300 | 4 | 1:50 |
| 80 | 42.D168 (EBOV-1) | 56 | 1:5000 | BMI300 | 2 | 1:50 |
| 81 | 3636.C75829.D28 | 4116 | 1:1 | NA | 4116 | 1:400 |
| 82 | 3636.C75829.D28 | 4116 | 1:3 | BMI300 | 1372 | 1:200 |
| 83 | Same as VTS 82 | 4116 | 1:3 | BMI300 | 1372 | 1:400 |
| 84 | Same as VTS 82 | 4116 | 1:3 | BMI300 | 1372 | 1:100 |
| 85 | 3636.C75829.D28 | 4116 | 1:9 | BMI300 | 457 | 1:50 |
| 86 | 3636.C75829.D28 | 4116 | 1:27 | BMI300 | 152 | 1:50 |
| 87 | 3636.C75829.D28 | 457 | 1:81 | BMI300 | 51 | 1:50 |
| 88 | 3636.C75829.D28 | 457 | 1:243 | BMI300 | 17 | 1:50 |
| 89 | 3636.C75829.D28 | 152 | 1:729 | BMI300 | 6 | 1:50 |
| 90 | 3636.C75829.D28 | 51 | 1:2187 | BMI300 | 2 | 1:50 |

**S6 Table. Preparation of Validation Test Samples (continued)**

| **VTS #** | **Test Specimen ID** | **Anti-GP IgG Concentration  (ELISA Units/mL) (Before Spike)** | **Final Dilution Factor  (Spike)** | **Negative Specimen Identifier  (Diluent)** | **Expected VTS Anti-GP IgG Concentration  (ELISA Units/mL)** | **VTS Starting Dilution** |
| --- | --- | --- | --- | --- | --- | --- |
| 91 | M06324.D35 (EBOV-3) | 4572 | 1:1 | NA | 4572 | 1:800 |
| 92 | Same as VTS 91 | 4572 | 1:1 | NA | 4572 | 1:1600 |
| 93 | Same as VTS 91 | 4572 | 1:1 | NA | 4572 | 1:400 |
| 94 | M06324.D35 (EBOV-3) | 4572 | 1:3 | BMI300 | 1524 | 1:200 |
| 95 | M06324.D35 (EBOV-3) | 4572 | 1:9 | BMI300 | 508 | 1:100 |
| 96 | M06324.D35 (EBOV-3) | 4572 | 1:27 | BMI300 | 169 | 1:50 |
| 97 | M06324.D35 (EBOV-3) | 508 | 1:81 | BMI300 | 56 | 1:50 |
| 98 | M06324.D35 (EBOV-3) | 508 | 1:243 | BMI300 | 19 | 1:50 |
| 99 | M06324.D35 (EBOV-3) | 169 | 1:729 | BMI300 | 6 | 1:50 |
| 100 | M06324.D35 (EBOV-3) | 56 | 1:2187 | BMI300 | 2 | 1:50 |
| 101 | 20.D112 (EBOV-1) | 5188 | 1:5 | BMI300 | 1038 | 1:100 |
| 102 | 20.D112 (EBOV-1) | 5188 | 1:5 | CYN179176 (Male) | 1038 | 1:100 |
| 103 | 20.D112 (EBOV-1) | 5188 | 1:5 | CYN179177 (Male) | 1038 | 1:100 |
| 104 | 20.D112 (EBOV-1) | 5188 | 1:5 | CYN179179 (Male) | 1038 | 1:100 |
| 105 | 20.D112 (EBOV-1) | 5188 | 1:5 | CYN179184 (Female) | 1038 | 1:100 |
| 106 | 20.D112 (EBOV-1) | 5188 | 1:5 | CYN179185 (Female) | 1038 | 1:100 |
| 107 | 20.D112 (EBOV-1) | 5188 | 1:50 | BMI300 | 104 | 1:50 |
| 108 | 20.D112 (EBOV-1) | 5188 | 1:50 | CYN179176 (Male) | 104 | 1:50 |
| 109 | 20.D112 (EBOV-1) | 5188 | 1:50 | CYN179177 (Male) | 104 | 1:50 |
| 110 | 20.D112 (EBOV-1) | 5188 | 1:50 | CYN179179 (Male) | 104 | 1:50 |
| 111 | 20.D112 (EBOV-1) | 5188 | 1:50 | CYN179184 (Female) | 104 | 1:50 |
| 112 | 20.D112 (EBOV-1) | 5188 | 1:50 | CYN179185 (Female) | 104 | 1:50 |
| 113 | M06957.D42 (EBOV-3) | 11201 | 1:5 | BMI300 | 2240 | 1:400 |
| 114 | M06957.D42 (EBOV-3) | 11201 | 1:5 | CYN179176 (Male) | 2240 | 1:400 |
| 115 | M06957.D42 (EBOV-3) | 11201 | 1:5 | CYN179177 (Male) | 2240 | 1:400 |
| 116 | M06957.D42 (EBOV-3) | 11201 | 1:5 | CYN179179 (Male) | 2240 | 1:400 |
| 117 | M06957.D42 (EBOV-3) | 11201 | 1:5 | CYN179184 (Female) | 2240 | 1:400 |
| 118 | M06957.D42 (EBOV-3) | 11201 | 1:5 | CYN179185 (Female) | 2240 | 1:400 |
| 119 | M06957.D42 (EBOV-3) | 11201 | 1:50 | BMI300 | 224 | 1:50 |

**S6 Table. Preparation of Validation Test Samples (continued)**

| **VTS #** | **Test Specimen ID** | **Anti-GP IgG Concentration  (ELISA Units/mL) (Before Spike)** | **Final Dilution Factor  (Spike)** | **Negative Specimen Identifier  (Diluent)** | **Expected VTS Anti-GP IgG Concentration  (ELISA Units/mL)** | **VTS Starting Dilution** |
| --- | --- | --- | --- | --- | --- | --- |
| 120 | M06957.D42 (EBOV-3) | 11201 | 1:50 | CYN179176 (Male) | 224 | 1:50 |
| 121 | M06957.D42 (EBOV-3) | 11201 | 1:50 | CYN179177 (Male) | 224 | 1:50 |
| 122 | M06957.D42 (EBOV-3) | 11201 | 1:50 | CYN179179 (Male) | 224 | 1:50 |
| 123 | M06957.D42 (EBOV-3) | 11201 | 1:50 | CYN179184 (Female) | 224 | 1:50 |
| 124 | M06957.D42 (EBOV-3) | 11201 | 1:50 | CYN179185 (Female) | 224 | 1:50 |
| 125 | 549.D63 (EBOV-2) | 31789 | 1:5 | BMI300 | 6358 | 1:800 |
| 126 | 549.D63 (EBOV-2) | 31789 | 1:5 | CYN179176 (Male) | 6358 | 1:800 |
| 127 | 549.D63 (EBOV-2) | 31789 | 1:5 | CYN179177 (Male) | 6358 | 1:800 |
| 128 | 549.D63 (EBOV-2) | 31789 | 1:5 | CYN179179 (Male) | 6358 | 1:800 |
| 129 | 549.D63 (EBOV-2) | 31789 | 1:5 | CYN179184 (Female) | 6358 | 1:800 |
| 130 | 549.D63 (EBOV-2) | 31789 | 1:5 | CYN179185 (Female) | 6358 | 1:800 |
| 131 | 549.D63 (EBOV-2) | 31789 | 1:50 | BMI300 | 636 | 1:100 |
| 132 | 549.D63 (EBOV-2) | 31789 | 1:50 | CYN179176 (Male) | 636 | 1:100 |
| 133 | 549.D63 (EBOV-2) | 31789 | 1:50 | CYN179177 (Male) | 636 | 1:100 |
| 134 | 549.D63 (EBOV-2) | 31789 | 1:50 | CYN179179 (Male) | 636 | 1:100 |
| 135 | 549.D63 (EBOV-2) | 31789 | 1:50 | CYN179184 (Female) | 636 | 1:100 |
| 136 | 549.D63 (EBOV-2) | 31789 | 1:50 | CYN179185 (Female) | 636 | 1:100 |
| 137 | 05400.53946.D10 | 63 | 1:1 | NA | 63 | 1:50 |
| 138 | 563.D10 (EBOV-2) | 72 | 1:1 | NA | 72 | 1:50 |
| 139 | 27.D7 (EBOV-1) | 75 | 1:1 | NA | 75 | 1:50 |
| 140 | 05400.43909.D10 | 79 | 1:1 | NA | 79 | 1:50 |
| 141 | 05400.43367.D10 | 81 | 1:1 | NA | 81 | 1:50 |
| 142 | 232.D10 (EBOV-2) | 101 | 1:1 | NA | 101 | 1:50 |
| 143 | 232.D14 (EBOV-2) | 127 | 1:1 | NA | 127 | 1:50 |
| 144 | 05400.05233.D270 | 133 | 1:1 | NA | 133 | 1:50 |
| 145 | 05400.69487.D330 | 187 | 1:1 | NA | 187 | 1:50 |
| 146 | 05400.43909.D28 | 225 | 1:1 | NA | 225 | 1:50 |
| 147 | 05400.69487.D399 | 277 | 1:1 | NA | 277 | 1:50 |
| 148 | 569.D10 (EBOV-2) | 307 | 1:1 | NA | 307 | 1:50 |
| 149 | 05400.43909.D301 | 324 | 1:1 | NA | 324 | 1:50 |

**S6 Table. Preparation of Validation Test Samples (continued)**

| **VTS #** | **Test Specimen ID** | **Anti-GP IgG Concentration  (ELISA Units/mL) (Before Spike)** | **Final Dilution Factor  (Spike)** | **Negative Specimen Identifier  (Diluent)** | **Expected VTS Anti-GP IgG Concentration  (ELISA Units/mL)** | **VTS Starting Dilution** |
| --- | --- | --- | --- | --- | --- | --- |
| 150 | 05400.08421.D178 | 416 | 1:1 | NA | 416 | 1:50 |
| 151 | 160.D10 (EBOV-2) | 433 | 1:1 | NA | 433 | 1:50 |
| 152 | 05400.08421.D148 | 568 | 1:1 | NA | 568 | 1:100 |
| 153 | M06957.D14 (EBOV-3) | 691 | 1:1 | NA | 691 | 1:100 |
| 154 | 8200.203R.D16 | 1301 | 1:1 | NA | 1301 | 1:200 |
| 155 | M06781.D14 (EBOV-3) | 1611 | 1:1 | NA | 1611 | 1:200 |
| 156 | 59.D14 (EBOV-1) | 2033 | 1:1 | NA | 2033 | 1:400 |
| 157 | 3636.C75977.D56 | 3109 | 1:1 | NA | 3109 | 1:400 |
| 158 | 16.D365 (EBOV-1) | 10323 | 1:1 | NA | 10323 | 1:1600 |
| 159 | M05520.D35 (EBOV-3) | 31461 | 1:1 | NA | 31461 | 1:3200 |
| 160 | 613.D49 (EBOV-2) | 178039 | 1:1 | NA | 178039 | 1:25600 |
| 161 | 4C94.D63 (EBOV-2) | 200619 | 1:1 | NA | 200619 | 1:25600 |
