# Supplementary material for: Method feasibility for cross-species testing, qualification, and validation of the Filovirus Animal Nonclinical Group anti-Ebola virus glycoprotein immunoglobulin G enzyme-linked immunosorbent assay for non-human primate serum samples
Source: PLoS One. 2020 Oct 29;15(10):e0241016. doi: 10.1371/journal.pone.0241016 (PMC7595334; doi:10.1371/journal.pone.0241016)
Supplement: S7 Table — (DOCX) [file pone.0241016.s010.docx]

**S7 Table. Plate Layout and Optical Density Results Used to Determine Conjugate Dilution**

|  | **Conjugate Dilution**  **(1:X)** | **NHP RS Dilution (1:X)** | | | | | | | | | | | |
| --- | --- | --- | --- | --- | --- | --- | --- | --- | --- | --- | --- | --- | --- |
|  |  | **110** | **220** | **441** | **883** | **1766** | **3532** | **7065** | **14131** | **28262** | **56524** | **113049** | **Blank** |
|  |  | **1** | **2** | **3** | **4** | **5** | **6** | **7** | **8** | **9** | **10** | **11** | **12** |
| **Plate 1, Operator 1** | | | | | | | | | | | | | |
| **A** | **4,000** | 3.433 | 3.399 | 3.395 | 3.331 | 3.072 | 2.172 | 1.274 | 0.682 | 0.350 | 0.184 | 0.096 | 0.035 |
| **B** | **6,000** | 3.399 | 3.368 | 3.368 | 3.242 | 2.579 | 1.620 | 0.920 | 0.447 | 0.282 | 0.120 | 0.084 | 0.041 |
| **C** | **8,000** | 3.390 | 3.378 | 3.353 | 3.012 | 2.172 | 1.247 | 0.674 | 0.349 | 0.186 | 0.095 | 0.055 | 0.039 |
| **D** | **10,000** | 3.351 | 3.296 | 3.242 | 2.565 | 1.747 | 0.912 | 0.509 | 0.258 | 0.179 | 0.077 | 0.384 | 0.014 |
| **E** | **12,000** | 3.342 | 3.346 | 3.138 | 2.405 | 1.488 | 0.851 | 0.446 | 0.223 | 0.445 | 0.153 | 0.054 | 0.032 |
| **F** | **14,000** | 3.319 | 3.313 | 3.084 | 2.381 | 1.555 | 0.88 | 0.356 | 0.244 | 0.165 | 0.064 | 0.078 | 0.011 |
| **G** | **16,000** | 3.385 | 3.269 | 3.000 | 2.244 | 1.318 | 0.699 | 0.361 | 0.187 | 0.109 | 0.048 | 0.042 | 0.024 |
| **H** | **Blank** | 0.003 | -0.023 | 0.012 | -0.023 | 0.011 | -0.017 | 0.024 | -0.011 | 0.027 | -0.02 | 0.042 | -0.015 |
| **Plate 2, Operator 1** | | | | | | | | | | | | | |
| **A** | **4,000** | 3.381 | 3.419 | 3.342 | 3.306 | 3.081 | 2.157 | 1.236 | 0.637 | 0.329 | 0.171 | 0.090 | 0.074 |
| **B** | **6,000** | 3.349 | 3.372 | 3.370 | 3.191 | 2.566 | 1.624 | 0.923 | 0.472 | 0.261 | 0.120 | 0.083 | 0.040 |
| **C** | **8,000** | 3.340 | 3.358 | 3.361 | 2.965 | 2.126 | 1.205 | 0.669 | 0.348 | 0.183 | 0.088 | 0.052 | 0.045 |
| **D** | **10,000** | 3.310 | 3.328 | 3.169 | 2.522 | 1.711 | 0.899 | 0.503 | 0.246 | 0.138 | 0.059 | 0.026 | 0.018 |
| **E** | **12,000** | 3.362 | 3.332 | 3.115 | 2.340 | 1.459 | 0.823 | 0.431 | 0.216 | 0.111 | 0.052 | 0.033 | 0.029 |
| **F** | **14,000** | 3.314 | 3.345 | 3.091 | 2.284 | 1.452 | 0.828 | 0.363 | 0.235 | 0.138 | 0.054 | 0.069 | 0.008 |
| **G** | **16,000** | 3.332 | 3.312 | 2.987 | 2.227 | 1.305 | 0.683 | 0.353 | 0.185 | 0.097 | 0.045 | 0.032 | 0.025 |
| **H** | **Blank** | 0.007 | -0.019 | 0.017 | 0.002 | 0.019 | -0.008 | 0.014 | -0.004 | 0.008 | -0.011 | 0.035 | -0.017 |

**S7 Table. Plate Layout and Optical Density Results Used to Determine Conjugate Dilution (continued)**

|  | | **Conjugate Dilution**  **(1:X)** | | **NHP RS Dilution (1:X)** | | | | | | | | | | | | | | | | | | | | | | |
| --- | --- | --- | --- | --- | --- | --- | --- | --- | --- | --- | --- | --- | --- | --- | --- | --- | --- | --- | --- | --- | --- | --- | --- | --- | --- | --- |
|  |  |  |  | **110** | | **220** | | **441** | | **883** | | **1766** | | **3532** | | **7065** | | **14131** | | **28262** | | **56524** | | **113049** | | **Blank** |
|  |  |  |  | **1** | | **2** | | **3** | | **4** | | **5** | | **6** | | **7** | | **8** | | **9** | | **10** | | **11** | | **12** |
|  | **Plate 3, Operator 2** | | | | | | | | | | | | | | | | | | | | | | | | | |
| **A** | **4,000** | | 3.430 | | 3.386 | | 3.373 | | 3.207 | | 2.652 | | 1.649 | | 0.921 | | 0.490 | | 0.257 | | 0.130 | | 0.074 | | 0.164 | |
| **B** | **6,000** | | 3.357 | | 3.340 | | 3.282 | | 2.860 | | 1.924 | | 1.159 | | 0.635 | | 0.321 | | 0.187 | | 0.079 | | 0.064 | | 0.014 | |
| **C** | **8,000** | | 3.353 | | 3.333 | | 3.188 | | 2.509 | | 1.677 | | 0.923 | | 0.515 | | 0.261 | | 0.134 | | 0.068 | | 0.045 | | 0.027 | |
| **D** | **10,000** | | 3.349 | | 3.273 | | 2.997 | | 2.110 | | 1.347 | | 0.706 | | 0.400 | | 0.196 | | 0.139 | | 0.052 | | 0.030 | | 0.016 | |
| **E** | **12,000** | | 3.312 | | 3.173 | | 2.739 | | 1.999 | | 1.190 | | 0.659 | | 0.373 | | 0.197 | | 0.102 | | 0.051 | | 0.034 | | 0.026 | |
| **F** | **14,000** | | 3.261 | | 3.023 | | 2.396 | | 1.636 | | 1.035 | | 0.563 | | 0.278 | | 0.177 | | 0.123 | | 0.046 | | 0.048 | | 0.010 | |
| **G** | **16,000** | | 3.210 | | 2.932 | | 2.350 | | 1.601 | | 0.903 | | 0.500 | | 0.252 | | 0.152 | | 0.085 | | 0.039 | | 0.029 | | 0.015 | |
| **H** | **Blank** | | 0.005 | | -0.016 | | 0.019 | | -0.006 | | 0.017 | | -0.005 | | 0.021 | | 0.000 | | 0.005 | | -0.010 | | 0.021 | | -0.014 | |
|  | **Plate 4, Operator 2** | | | | | | | | | | | | | | | | | | | | | | | | | |
| **A** | **4,000** | | 3.391 | | 3.329 | | 3.355 | | 3.228 | | 2.773 | | 1.691 | | 0.914 | | 0.495 | | 0.259 | | 0.131 | | 0.071 | | 0.058 | |
| **B** | **6,000** | | 3.385 | | 3.327 | | 3.238 | | 2.894 | | 1.999 | | 1.204 | | 0.640 | | 0.317 | | 0.180 | | 0.073 | | 0.148 | | 0.031 | |
| **C** | **8,000** | | 3.374 | | 3.376 | | 3.228 | | 2.587 | | 1.700 | | 0.943 | | 0.498 | | 0.256 | | 0.133 | | 0.063 | | 0.038 | | 0.038 | |
| **D** | **10,000** | | 3.353 | | 3.257 | | 3.085 | | 2.148 | | 1.386 | | 0.713 | | 0.410 | | 0.201 | | 0.144 | | 0.044 | | 0.028 | | 0.010 | |
| **E** | **12,000** | | 3.339 | | 3.238 | | 2.851 | | 2.130 | | 1.234 | | 0.661 | | 0.368 | | 0.184 | | 0.105 | | 0.046 | | 0.034 | | 0.027 | |
| **F** | **14,000** | | 3.272 | | 3.059 | | 2.508 | | 1.796 | | 1.035 | | 0.568 | | 0.267 | | 0.174 | | 0.131 | | 0.036 | | 0.050 | | 0.008 | |
| **G** | **16,000** | | 3.226 | | 2.994 | | 2.424 | | 1.631 | | 0.935 | | 0.514 | | 0.269 | | 0.146 | | 0.081 | | 0.036 | | 0.026 | | 0.018 | |
| **H** | **Blank** | | 0.005 | | -0.022 | | 0.023 | | -0.008 | | 0.018 | | -0.008 | | 0.024 | | 0.004 | | 0.019 | | -0.011 | | 0.034 | | -0.015 | |
